# Assessing the therapeutic potential of PDE‐5 inhibitors in adults: Insights from a multi‐omics study

**DOI:** 10.14814/phy2.71104

**Published:** 2026-09-21

**Authors:** Kristen A. McGovern, Traver J. Wright, Erik D. Marchant, Sean P. Kilroe, William J. Durham, E. Lichar Dillon, Marc M. Baum, Michael P. Kinsky, William K. Russell, Christopher S. Fry, Pratik Gongloor, Richard B. Pyles, Randall J. Urban, Blake B. Rasmussen, Melinda Sheffield‐Moore

**Affiliations:** ^1^ Department of Internal Medicine The University of Texas Medical Branch (UTMB) Galveston Texas USA; ^2^ Department of Biological Sciences Ohio University Athens Ohio USA; ^3^ Department of Cellular and Integrative Physiology University of Texas Health Science Center at San Antonio San Antonio Texas USA; ^4^ Department of Chemistry Oak Crest Institute of Science Monrovia California USA; ^5^ Department of Biochemistry and Molecular Biology UTMB Galveston Texas USA; ^6^ Department of Athletic Training and Clinical Nutrition University of Kentucky Lexington Kentucky USA; ^7^ John Sealy School of Medicine UTMB Galveston Texas USA; ^8^ Department of Pediatrics UTMB Galveston Texas USA

**Keywords:** arginine, eicosanoids, multi‐omics, phosphodiesterase‐5 inhibitor, sildenafil, tadalafil

## Abstract

Phosphodiesterase‐5 inhibitors have beneficial pleiotropic effects and hold promise as adjuvant treatments for cancer, dementia, and other disorders. Sixteen men and women aged 50–60 were administered daily sildenafil/tadalafil for 1 month. We characterized molecular signatures of sildenafil/tadalafil treatment via analyses and multi‐omics integration of the serum metabolome and skeletal muscle proteome and transcriptome. In serum, eicosanoids decreased and arginine biosynthesis pathway components increased following sildenafil/tadalafil. In skeletal muscle following sildenafil, blood vessel development and immune response genes increased, indicating augmented angiogenesis and possible inflammation at proximate biopsy sites. Proteins related to telomere maintenance and protein folding increased, suggesting reduced cellular senescence and cellular stress or functional adaptations. In skeletal muscle following tadalafil, there were no differentially expressed genes, and despite reduction of many proteins there were no enriched biological processes. Notably, sildenafil resulted in a more cohesive and substantial molecular signal in skeletal muscle. Multi‐omics integration for both treatments revealed significant coexpression patterns. Phosphodiesterase‐5 inhibitors showed systemic anti‐inflammatory effects, as indicated by reduced serum levels of pro‐inflammatory eicosanoids. However, this did not translate to immunohistochemical changes. Additional research is warranted to investigate PDE‐5 inhibitor effects on various tissues, which can inform prospective applications for drug repurposing.

## 1 INTRODUCTION

Phosphodiesterase‐5 (PDE‐5) inhibitors such as sildenafil and tadalafil enhance nitric oxide (NO)‐mediated vasodilation (Dishy et al., 2001) and are prescribed primarily for the treatment of erectile dysfunction and pulmonary arterial hypertension (Tulis & Middlemas, 2024). PDE‐5 inhibitors amplify protein kinase G (PKG) signaling by impairing degradation of cyclic guanosine monophosphate (cGMP). PKG signaling regulates a variety of processes related to cellular health, including calcium signaling and reactive oxygen species (ROS) production. By impacting central cellular processes such as these, PDE‐5 inhibitors have broad effects in a variety of cells and tissues (Pușcașu et al., 2023).

A number of studies suggest PDE‐5 inhibitors impact muscle physiology and exercise endurance, although the specific cellular response mechanisms involved remain uncertain (Martin et al., 2012; Percival et al., 2012; Winn et al., 2025). In a previous study, we found that short‐term (8‐days) daily administration of 25 mg sildenafil increased muscle protein synthesis and reduced muscle fatigue in healthy adult males, indicating that it could directly enhance skeletal muscle function (Sheffield‐Moore et al., 2013). Over the past decade, other investigators seeking to repurpose these drugs have discovered additional effects beyond their vasodilatory properties. Research suggests that PDE‐5 inhibitors have the potential to aid in treatment for conditions such as cancer (Cruz‐Burgos et al., 2021), COVID‐19 (Varghese et al., 2023), and dementia (Hainsworth et al., 2023), while ameliorating features common to many conditions, including inflammation (Di Luigi et al., 2020; Giannattasio et al., 2019; Santi et al., 2016), endothelial dysfunction (Aversa et al., 2008; Halcox et al., 2002; Rodriguez‐Miguelez et al., 2018), and oxidative stress (Zhao et al., 2011).

To better characterize the general and muscle‐specific responses to PDE‐5 inhibitors, we administered daily sildenafil or tadalafil to healthy adult men and women in an 8‐week randomized, double‐blind crossover study. In the study, PDE‐5 inhibitors were given for 4 weeks, and placebos were given for 4 weeks. As our research interests overlap significantly with the reported pleiotropic effects of PDE‐5 inhibitors, particularly their potential to improve inflammation, skeletal muscle function, and cognition (Burckart et al., 2010; Durham et al., 2009; Wright, Elliott, et al., 2024; Wright, Sheffield‐Moore, et al., 2024), we conducted exploratory analyses of PDE‐5 inhibitor effects on skeletal muscle and serum samples. Samples were analyzed using proteomic, metabolomic, transcriptomic, and immunohistochemical approaches with the aim of gaining additional insights into the cellular and systemic consequences of PDE‐5 inhibition.

## 2 METHODS

We conducted a randomized, double‐blinded crossover study, which was approved by The University of Texas Medical Branch (UTMB) Institutional Review Board (12‐153) and registered at www.clinicaltrials.gov (NCT01661595) before recruitment. All subjects provided written informed consent prior to enrollment. Data were collected in accordance with the Declaration of Helsinki. Subjects were recruited from the local community using flyers and local postings.

Subjects were eligible if they were between the ages of 50–60 years with stable body weight for the previous 3 months. Subjects were excluded if they had significant heart, liver, kidney, blood or respiratory disease, peripheral vascular disease, untreated diabetes mellitus or other endocrine disease, active cancer, use of alpha blockers or nitrates, treatment with anabolic steroids, or corticosteroids within the previous 6 months, current alcohol or drug abuse, cardiac abnormalities, pulmonary hypertension, severe depression, systolic blood pressure <100 or >150, diastolic blood pressure <60 or >90, impairments in any Activities of Daily Living (ADLs), history of falls (≥2 per year), or significant weight loss in the past year.

The initial screening assessments included echocardiogram, electrocardiogram, urinalysis, and blood tests including fasting blood glucose, comprehensive metabolic panel, complete blood count, lipid panel, drug screening, Hepatitis B, Hepatitis C, and HIV. Subjects with possible indication of right‐to‐left interatrial shunt on the echocardiogram were cleared for enrollment if they passed an exercise stress test.

Enrolled subjects participated in an 8‐week crossover study that consisted of 4 weeks of treatment with one of two PDE‐5 inhibitors (sildenafil 50 mg/day or tadalafil 10 mg/day, taken orally once per day) as well as 4 weeks of visually identical placebo taken orally once per day. Subjects were randomized to one of four groups receiving treatment or placebo in random order without blocking (Table 1). Subjects and investigators were blinded to treatments, which were compounded and dispensed by the UTMB Investigational Drug Service research pharmacy. Placebo capsules comprised size 0 red gel caps (Health Care Logistics) filled with lactose monohydrate powder (J.T. Baker or Letcos Medical). Treatment capsules were identical in appearance but included a sildenafil (50 mg, halved to fit in capsule, NDC# 00069‐4210‐30) or tadalafil (10 mg, whole, NDC# 00002‐4463‐30) tablet. Subjects reported to the UTMB Clinical Research Center (CRC) for testing at screening and study visits at weeks 0, 4, and 8. For each study visit, subjects arrived at the CRC the evening prior. Subjects were served a meal and snack at 22:00 and then fasted through the duration of the study visit the following day.

**TABLE 1:** Crossover study group descriptions.

Treatment sequence	Weeks 1–4	Weeks 5–8
Sildenafil‐Placebo (S‐P)	50 mg sildenafil per day (1 capsule)	1 placebo capsule per day
Tadalafil‐Placebo (T‐P)	10 mg tadalafil per day (1 capsule)	1 placebo capsule per day
Placebo‐Sildenafil (P‐S)	1 placebo capsule per day	50 mg sildenafil per day (1 capsule)
Placebo‐Tadalafil (P‐T)	1 placebo capsule per day	10 mg tadalafil per day (1 capsule)

**FIGURE 1:**
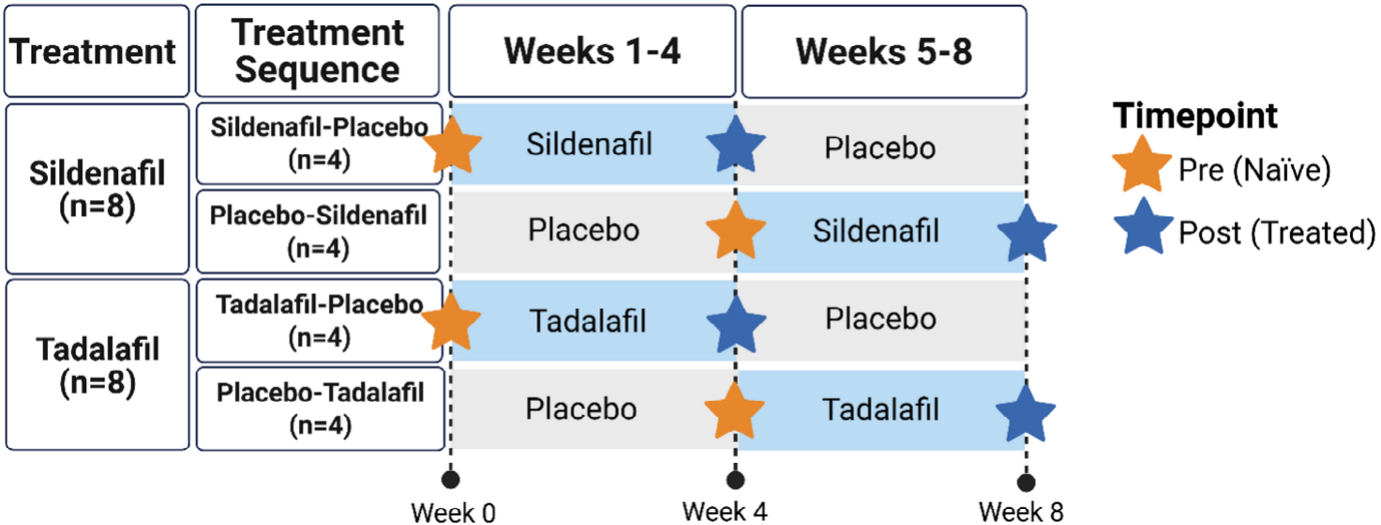
Crossover study design with pre‐treatment (naïve) and post‐treatment (treated) timepoint designations. Created with BioRender.

To ease interpretability, we simplified the analyses by comparing samples/measurements taken just prior to initiating PDE‐5 inhibitor treatment (Pre/ Naïve) to those taken following 4 weeks of PDE‐5 inhibitor treatment (Post/ Treated) (Figure 1). All Pre vs. Post analyses compare these two relative timepoints for the sildenafil (*n* = 8) and tadalafil (*n* = 8) groups. During each study visit, the subjects withheld their allotted daily treatment pill until the time designated in the study day timeline (Figure S1). During weeks 0 and 4, subjects ingested pills corresponding to their assigned treatment for weeks 1–4. During week 8, subjects ingested pills corresponding to their assigned treatment for weeks 5–8. Measurements and samples taken prior to pill ingestion were considered Pre‐Pill and those taken post pill ingestion were considered Post‐Pill.

### 2.1 Blood and skeletal muscle sampling

Venous blood samples were taken at the beginning of each study visit (pre‐pill) as well as 1 h post pill ingestion. Skeletal muscle biopsies (~50–200 mg) were taken from the vastus lateralis, 10–15 cm above the knee after numbing with 10 mL of 1% lidocaine. The study visit order and timing of events was similar but not identical during the week 0 study visit compared to study visits during weeks 4 and 8, which were identical (Figure S1). During the week 0 study visit, there were two muscle biopsies obtained, while one was obtained during the week 4 and week 8 study visits. Both muscle biopsies collected during the week 0 study visit were collected prior to pill ingestion. Tissue from the first biopsy was used for transcriptomics and immunohistochemistry, while tissue from the second biopsy was used for proteomics. Tissue from the solo biopsy during weeks 4 and 8 was used for transcriptomics, immunohistochemistry, and proteomics. Muscle biopsies were obtained from the same leg during all study visits with the exception of one subject from the tadalafil group, whose pre‐ and post‐ biopsies were taken from opposite legs. Diet was controlled, as subjects fasted from 10 pm the night prior through the study visit. Physical activity was similar among study visit days.

### 2.2 Sildenafil and tadalafil pharmacokinetics

Pre‐pill and 1‐h post‐pill plasma samples collected at each of the study visits were analyzed using LC‐MS/MS to measure concentrations of sildenafil, its metabolite *N*‐desmethyl sildenafil, as well as tadalafil and its metabolite *N‐*desmethyl tadalafil. An analytical standard could not be obtained for methylcatechol glucuronide, the main metabolite of tadalafil (Mehrotra et al., 2007), so a decision was made to instead measure the metabolite *N*‐desmethyl tadalafil. Pre‐pill plasma samples were taken ~5 h prior to pill ingestion during the week 0 study visit and ~2 h prior to pill ingestion during the weeks 4 and 8 study visits. Naïve human plasma (li‐heparin, Innovative Research, IPLAWBLIH) was purchased from Innovative Research (Novi, MI) for use as blanks and for standard curve preparation for each of the four analytes. Standard curves for sildenafil (Sigma‐Aldrich, PHR1807), *N*‐desmethyl sildenafil (Santa Cruz Biotechnology, sc‐208029) and tadalafil (Santa Cruz Biotechnology, sc‐208412) were obtained using standard concentrations of 0.5, 1, 3, 10, 100, 250, and 500 ng/mL, while the standard curve for *N*‐desmethyl tadalafil (Santa Cruz Biotechnology, sc‐500690) was prepared using standard concentrations of 0.5, 1, 5, 10, 15, 50, 75, and 100 ng/mL. Quality controls with concentrations of 5, 25, and 50 ng/mL for each analyte were run (in duplicate, at minimum) to determine percent relative error. Sildenafil‐d8 (Santa Cruz Biotechnology, sc‐472647) and tadalafil‐d3 (Santa Cruz Biotechnology, sc‐473933) were used as internal standards. Bioanalytical methods were developed in accordance with FDA guidelines (U.S. Department of Health and Human Services Food and Drug Administration et al., 2018).

Plasma samples were processed using a protein and phospholipid removal system (Phree; Phenomenex, Inc., Torrance, CA) according to the manufacturer's instructions with 1% vol/vol formic acid‐acetonitrile as the elution buffer, and reconstituted with 0.1% vol/vol formic acid and 10% vol/vol acetonitrile in water prior to analysis. Sample analysis by Liquid Chromatography‐Mass Spectrometry (LC‐MS/MS) was performed using an 1100 series HPLC system (Agilent Technologies, Santa Clara, CA) interfaced to an API‐3000 triple‐quadrupole tandem mass spectrometer (AB Sciex, Framingham, MA) with a turbo ion spray electrospray ionization (ESI) source. A Waters (Milford, MA) Atlantis T3 column (2.1 × 50 mm, 5 μm) controlled at 40°C was the stationary phase. The following gradient program was used (A, 0.1%, vol/vol, formic acid in water; B, 0.1%, vol/vol, formic acid in acetonitrile): 0.5 min of 10% B, 2‐min ramp from 90:10 A:B to 30:70 A:B, 0.4‐min ramp from 30:70 A:B to 0:100 A:B, 1‐min hold at 0:100 A:B, 0.1‐min ramp from 0:100 A:B to 90:10 A:B, and 2.59‐min hold at 90:10 A:B. The program resulted in a total run time of 6 min. The measured transition ions, m/z, under ESI+ ionization mode were the following: sildenafil, 475.2 amu (parent) and 283.3 amu (product); *N*‐desmethyl sildenafil, 461.2 amu (parent) and 283.3 amu (product); tadalafil, 390.4 amu (parent) and 268.4 amu (product); *N*‐desmethyl tadalafil, 376.3 amu (parent) and 254.4 amu (product); sildenafil‐d8 (IS), 483.4 amu (parent) and 62.2 amu (product); and tadalafil‐d3 (IS), 393.4 amu (parent) and 271.3 amu (product). The representative lower limits of quantification (LLOQs) for the analytes were as follows: sildenafil (3.7 ng/mL); *N*‐desmethyl sildenafil (1.4 ng/mL); tadalafil (14 ng/mL); *N*‐desmethyl tadalafil (2.4 ng/mL). Estimated concentrations falling below LLOQs are reported; LLOQs are clearly delimited on graphs.

### 2.3 Plasma metabolomics

Pre‐pill plasma samples collected at each of the study visits were analyzed for lipid mediators and arginine metabolites. These metabolites were extracted from 50 μL of plasma by the addition of 400 μL methanol protein precipitation. Extractions were vortexed and incubated for 10 min at 4°C before being centrifuged at 17,000 × g to pellet protein and other debris. Supernatants were transferred to new tubes and dried under a stream of nitrogen gas. Dried extracts were resuspended in 50% methanol containing 0.01% butylated hydroxytoluene to prevent oxidation and then prepared for LC‐MS/MS analysis. Control extractions included a solvent only extraction and an internal standard (IS) only extraction. Prior to extraction, a mix of isotopically labeled lipid mediators, arginine, citrulline, proline, and ornithine were added as internal standards. Analysis by LC‐MS/MS was performed using a 1260 Infinity UHPLC System (Agilent) coupled to a QTRAP 6500 mass spectrometer (SCIEX) via Multiple Reaction Monitoring (MRM). Quality controls (QC) included a pooled QC prepared by mixing an aliquot of each sample into a single autosampler vial. Injection of the pooled QC every 8 sample injections monitored for changes in signal intensity and retention time drift. System suitability QC contained a mix of lipid mediators and were injected before and after the sample queue to monitor for system performance. Lipid mediators and arginine metabolites were separated by reverse‐phase chromatography on a Kinetex EVO C18 column (2.6 μm, 150 × 2.1 mm, Phenomenex). Mobile phases included: (A) water +0.1% formic acid, (B) acetonitrile +0.1% formic acid. The mobile phase gradient was as follows: 10% B from 0 to 3 min, 10%–30% B from 3 to 6 min, 30%–70% B from 6 to 20 min, 70%–95% B from 20 to 23 min, 95% B from 23 to 27 min, 95%–10% B from 27 to 27.1 min, and 10% B from 27.1 to 34 min. The column temperature was held at 45°C and 5 μL were injected. LC/MS peaks were integrated using MultiQuant software (SCIEX). Peak areas were exported and normalized to the peak area of a representative IS using a custom R script to give relative values of “normalized area”. Lipid mediators with peak areas less than 1e4 in at least 1/3 of the samples were filtered from the final data set. The measured transition ions, m/z, of the final metabolites are listed in Table S1.

### 2.4 Skeletal muscle transcriptomics

Pre‐pill muscle samples collected before and after active treatment (weeks 0 and 4 for subjects assigned to PDE‐5 inhibitors for weeks 1–4 and weeks 4 and 8 for subjects assigned to PDE‐5 inhibitors for weeks 5–8) were subjected to RNA sequencing. Approximately 20 mg of each sample was added to 300 μL Roche external lysis buffer (Roche Diagnostics, Indianapolis, IN) in a 0.1 glass Powerbead tube (Qiagen, Germantown MD). The sample tube was shaken in a Qiagen Tissue Lyser at 30 Hz for 5 min, then centrifuged at 13,000 × *g* for 1 min in a benchtop microcentrifuge. The homogenized tissue lysates were deposited into individual wells of 96 deep‐well processing plates (Roche Diagnostics). Nucleic acids were extracted in high‐throughput fashion using a MagNAPure 96 instrument employing large‐volume Cellular RNA extraction kits (Roche Diagnostics) according to the manufacturer's protocol for fresh/frozen biological samples. After extraction, a portion of the extracted RNA was immediately converted to complementary DNA (cDNA), and the remaining material was evaluated using RNA‐seq.

RNA‐seq evaluation was performed on extracted RNA using the Smart‐3SEQ method (Foley et al., 2019). Sample libraries were sequenced on an AVITI platform (Element Biosciences, San Diego, CA). Processed reads were aligned using STAR (Dobin et al., 2013) to hg38 gencode v40 reference. Reads per gene were then used to generate a composite count table used in downstream comparative analysis.

### 2.5 Skeletal muscle proteomics

Pre‐pill muscle samples collected at each of the study visits were subjected to label‐free quantitative proteomics analysis. Proteomics was performed essentially as described previously (Cabello et al., 2024; Granchi et al., 2026). Briefly, proteins were digested using S‐Trap columns (protifi.com) per manufacturer's instructions. Tryptic peptides were analyzed by nanoLC‐MS/MS (UltiMate 3000 RSLCnano–Orbitrap Eclipse) with FAIMS Pro (ICS v3.4; Standard Resolution; 100°C electrodes; Compensation Voltages (CVs) −35/−55/−75, 2 s each). Data Dependent Acquisition (DDA) used 120 k MS1 with ion‐trap MS2 (1.5 s cycle per CV). Peptides were separated on an Aurora column (75 μm × 25 cm, 1.6 μm) at 400 nL/min using 0.1% formic acid (FA) in water (A) and 0.1% FA in acetonitrile (B), with a ~140 min run (3% B hold then ramp to 28% B by ~101 min, to 38% by ~119 min, then wash at 90% B and re‐equilibrate).

Spectra were processed in Proteome Discoverer v2.2 and searched using Sequest against UniProt human with 5 ppm precursor/0.60 Da fragment tolerances, trypsin (≤2 missed cleavages), fixed carbamidomethyl (C), and variable oxidation (M) and deamidation.

IDs were validated in Scaffold 5.2.2 (peptides >95% probability; proteins >99% with ≥2 peptides; Protein Prophet) and exported via Scaffold DDA v6.7.2 at peptide/protein false discovery rate (FDR) <10% (q‐values). Indistinguishable proteins were grouped by parsimony and clustered when shared‐peptide evidence was moderate. Quantification used precursor intensities; missing values were imputed by QRILC and intensities normalized. For downstream analyses, the first protein in each cluster (by spectrum count) was used as the representative; gene symbols/names are reported.

### 2.6 Skeletal muscle immunohistochemistry

Immunohistochemistry was performed on pre‐pill muscle samples collected at the weeks 0, 4, and 8 study visits to quantify macrophage subtypes. A portion of skeletal muscle was set in Optimum Cutting Temperature (OCT) compound on cork and frozen in isopentane cooled in liquid nitrogen. Seven 7 μm‐thick sections were cut on a cryostat and allowed to air dry for approximately 30 min. The sections were then fixed at −20°C for 3 min in ice cold acetone and subsequently rehydrated in phosphate‐buffered saline (PBS). All washing steps described were performed with PBS. Endogenous peroxidases were blocked with 3% hydrogen peroxide for 8 min at room temperature (RT). The slides were washed and then blocked at RT with a streptavidin/biotin blocking kit (Vector Laboratories, SP‐2002), and then with 2.5% normal horse serum (NHS, ThermoFisher, 16050122) overnight at 4°C. The sections were then incubated overnight at 4°C in 2.5% NHS with Mouse Anti‐Human CD11b monoclonal antibody (Ab) (1:100; Cell Sciences, MON1019‐1) and wheat germ agglutinin AF647 (1:50; ThermoFisher, W32466). The slides were washed and then incubated for 90 min at RT in 2.5% NHS with Biotinylated Goat Anti‐Mouse IgG1 polyclonal Ab (1:1000; Jackson ImmunoResearch, 115‐065‐205). The slides were washed and incubated with a streptavidin‐horseradish peroxidase conjugate (included with kit) for 60 min at RT, washed, and incubated for 5 min at RT in AF488 Tyramide SuperBoost (ThermoFisher, kit B40932) according to the manufacturer's instructions. The slides were washed and blocked again for 1 h in 2.5% NHS at RT, followed by overnight incubation at 4°C in 2.5% NHS with Goat Anti‐Human CD206 polyclonal Ab (1:200; R&D Systems, AF2534). The slides were washed and then incubated for 90 min at RT in 2.5% NHS with Biotinylated Rabbit Anti‐Goat IgG Ab (1: 500; Vector Laboratories, BA‐5000‐1.5). Slides were washed and incubated for 60 min at RT in streptavidin‐Texas Red conjugate (1:200 in PBS; Vector Laboratories, SA‐5006‐1). The slides were washed and incubated for 10 min at RT in DAPI (1:10,000 in PBS; ThermoFisher, D3571). Slides were again washed and coverslips were mounted with VECTASHIELD Antifade Mounting Medium (Vector Laboratories, H‐1000‐10). The slides were imaged using a 20× objective on a Nikon Eclipse Ti inverted microscope. Macrophages were counted manually by a single person who was blinded to subject number and treatment group. M1 macrophages (CD11b+, CD206‐, DAPI+) and M2 macrophages (CD11b+, CD206+, DAPI+) were normalized to total muscle fiber count.

### 2.7 Statistical analyses

#### 2.7.1 Study demographics

Graphpad Prism 10.4.1 was used to compare demographics between treatment groups. Welch's ANOVA tests were used for continuous variables and Fisher's exact tests were used for categorical variables.

#### 2.7.2 Metabolomics

Missing values were imputed in R using the missForest algorithm (Stekhoven & Bühlmann, 2011). Statistical analyses were conducted using Metaboanalyst 6.0 (Pang et al., 2024) on paired pre‐pill samples from pre‐treatment (week 0 for S‐P/T‐P groups, and week 4 for P‐S/P‐T groups) and post‐treatment (week 4 for S‐P/T‐P groups, and week 8 for P‐S/P‐T groups) study visits. Metabolites determined to be near‐constant over time based on the interquartile range (IQR) estimate were filtered from the data set (cutoff = 5% of total metabolite number). Data were log2‐transformed and Pareto scaled before fitting limma‐based linear models with ANOVA‐style contrasts (Smyth, 2005). Models were fit separately for tadalafil and sildenafil. Subject was modeled as a fixed effect and raw *p* values (*α* = 0.05) were used to determine significant differential metabolites (Pang et al., 2022).

#### 2.7.3 Transcriptomics

Genes with <5 counts per million (cpm) for >90% of samples were filtered from the dataset. Differential expression analysis and testing for overrepresentation of gene ontology (GO) Biological Processes terms were completed in R following the edgeR quasi‐likelihood pipeline (Chen et al., 2016); subject was included as a factor in the design matrix to appropriately model the paired nature of the data (Chen et al., 2024). These analyses were completed separately for sildenafil and tadalafil and were conducted on count data from paired pre‐pill samples collected at the pre‐treatment (week 0 for S‐P/T‐P groups, and week 4 for P‐S/P‐T groups) and post‐treatment (week 4 for S‐P/T‐P groups, and week 8 for P‐S/P‐T groups) study visits. The FDR for differentially expressed genes (DEGs) was controlled using the Benjamini and Hochberg method with a cutoff of 0.1. GO terms with *p* values <10e‐5 were retained. To simplify interpretation of GO terms, a treemap was generated in R using the rrvgo package (Sayols, 2023), which clusters GO terms based on semantic similarity (threshold used = 0.7) and organizes them into nested rectangles. The largest GO term (based on total number of genes) in each cluster serves as the cluster representative inside which the other GO terms are nested. The size of each rectangle in the visualization is proportional to the p‐value associated with that term, with larger rectangles corresponding to more significant terms. Font size is automatically adjusted in the visualization to best fit within the dimensions of the corresponding rectangle. Gene ratios for enriched terms and pathways were calculated by dividing the number of genes associated with the pathway/term that were differentially expressed by the total number of genes associated with the pathway/term in the background gene set as summarized in the edgeR goana output. Heatmaps depicting mapping of DEGs to enriched GO Terms were constructed using code adapted from the R GOplot package (Walter et al., 2015). DEG‐GO associations were determined using the EnrichmentBrowser package (Geistlinger et al., 2016); it is noted that there are some annotation differences between EnrichmentBrowser and edgeR goana.

#### 2.7.4 Proteomics

Paired *t*‐tests were conducted using Scaffold DDA (version 6.7.2, Proteome Software, Inc.) on pre‐pill samples from pre‐treatment (week 0 for S‐P/T‐P groups, and week 4 for P‐S/P‐T groups) and post‐treatment (week 4 for S‐P/T‐P groups, and week 8 for P‐S/P‐T groups) study visits. Analyses were conducted separately for sildenafil and tadalafil. Raw *p* values (*α* = 0.05) were used to determine differentially expressed proteins. ShinyGO 0.82 (Ge et al., 2019) was used to determine the top 20 enriched GO Biological Processes terms with an FDR cutoff of 0.05. Fold enrichment for each term/pathway was calculated by dividing the percentage of differentially expressed proteins that are in the term/pathway by the corresponding percentage of the background proteins (log2‐fold change (logFC) of the differential proteins are not used in ShinyGO calculations). A list of all detected proteins in the samples was used as the background (reference) proteome. To simplify interpretation of GO terms, treemaps and heatmaps were generated in R using the same methods detailed above for transcriptomics. Reported logFC values were calculated in Scaffold DDA using summarization by timepoint.

#### 2.7.5 Multi‐omics integration

The Data Integration Analysis for Biomarker Discovery using Latent Variable Approaches for Omics Studies (DIABLO) method was implemented for each treatment using the mixOmics package in R to integrate the metabolomic, transcriptomic, and proteomic datasets. DIABLO constructs multiblock sparse partial least squares discriminant analysis (block‐sPLSDA) models that integrate multiple datasets (blocks) with the simultaneous aims of maximizing covariance among blocks and discriminating among categorical groups (Lê Cao & Welham, 2021; Rohart et al., 2017). Proteins with >20% missing data were removed from the protein block. Remaining missing values in the proteomics block were imputed using the Nonlinear Iterative Partial Least Squares (NIPALS) algorithm. Within‐subject variation was extracted from the normalized data using the within Variation function; these values were input into the block‐sPLSDA model, which applies centering and scaling to all variables (creates z‐scores). Weights of 0.1 in the design matrix prioritized variable selection toward discrimination over correlation. To prevent overfitting due to the small sample sizes, we specified that 10 variables be selected for each block on each component, and for interpretability we specified one component per block. The outputs of graphical functions from mixOmics, including loading plots on the component for each block, circos plots of inter‐block variable correlations with ≥ absolute value of 0.8, and heatmaps and associated dendrograms (hierarchical clustering on block variable *z*‐scores using Euclidean distance and complete linkage) were saved as objects and the data used to construct a single figure for each treatment using the circlize package in R (Gu et al., 2014).

#### 2.7.6 Immunohistochemistry

Paired *t*‐tests were conducted using Graphpad Prism 10.6.1 to compare pre‐treatment (week 0 for S‐P/T‐P groups, and week 4 for P‐S/P‐T groups) and post‐treatment (week 4 for S‐P/T‐P groups, and week 8 for P‐S/P‐T groups) values for M1, M2, and total macrophage counts. The Benjamin, Krieger, and Yekutieli method was used to control the false discovery rate (FDR, 0.1). Due to indications that there was an effect of time on counts (counts increased over time during placebo, Figure S12), in addition to a large amount of missing data, mixed models for repeated measures were fit in SAS with the following fixed effects: week 0 measurement, sequence, period, treatment, and week 0 measurement * period interaction (Mehrotra, 2014).

Additional methods are reported in the supplement.

## 3 RESULTS

### 3.1 Study subjects

**FIGURE 2:**
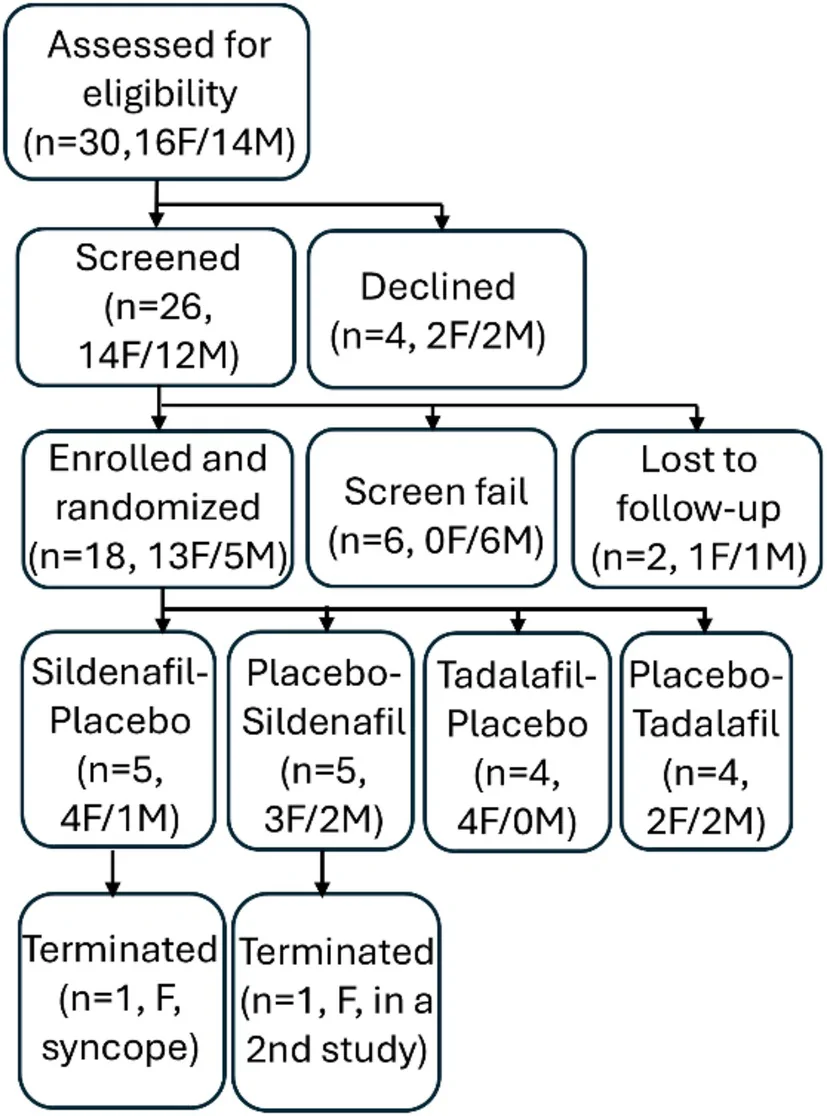
CONSORT diagram representing subject enrollment over the course of the study. Treatment sequences are named according to treatment order, for example, the Sildenafil‐Placebo group was administered sildenafil for weeks 1–4 and placebo for weeks 5–8.

Thirty potential participants were assessed for study eligibility (Figure 2). Four individuals declined to proceed with screening, six individuals failed the screening, and two individuals were lost to follow‐up. Eighteen subjects were enrolled and randomized to one of the four treatment sequences. One subject was terminated from the study during week 6 due to participation in a conflicting study. Another subject was terminated from the study after experiencing syncope. The data from the two terminated subjects were excluded from the analyses. Sixteen subjects completed the full 8‐week study.

The 16 subjects that completed the study averaged 54.9 ± 3.2 years of age and included 11 females and 5 males (Table 2). The pooled sildenafil group included 5 females and 3 males, and the pooled tadalafil group had 6 females and 2 males. The mean age of the sildenafil group was significantly younger than the tadalafil group. The mean Body Mass Index (BMI) for the tadalafil group fell just over the line into the “obese” category (>30 BMI), while the mean BMI for the sildenafil group fell just under in the “overweight” category. Mean BMI was not significantly different between groups.

**TABLE 2:** Study demographics.

Treatment	Sex (female/male)	Race/ethnicity (black/Caucasian/Hispanic)	Age (± SD)	BMI (± SD)
Sildenafil	5/3^A^	0/7/1^A^	53.3 ± 3.2^A^	29.8 ± 5.6^A^
Tadalafil	6/2^A^	1/4/3^A^	56.6 ± 2.3^B^	30.3 ± 3.1^A^
Total	11/5	1/11/4	54.9 ± 3.2	30.1 ± 4.7

*Note*: Means and counts with different letters (by column) are significantly different. Adjusted *p* value for age comparison sildenafil to tadalafil was 0.034.

### 3.2 Pharmacokinetics

Treatment‐naïve measurements were taken during week 0 for the S‐P and T‐P treatment sequence groups. Treated measurements were taken during week 4 for the S‐P and T‐P treatment sequence groups, and during week 8 for the P‐S and P‐T treatment sequence groups.

#### 3.2.1 Sildenafil

**FIGURE 3:**
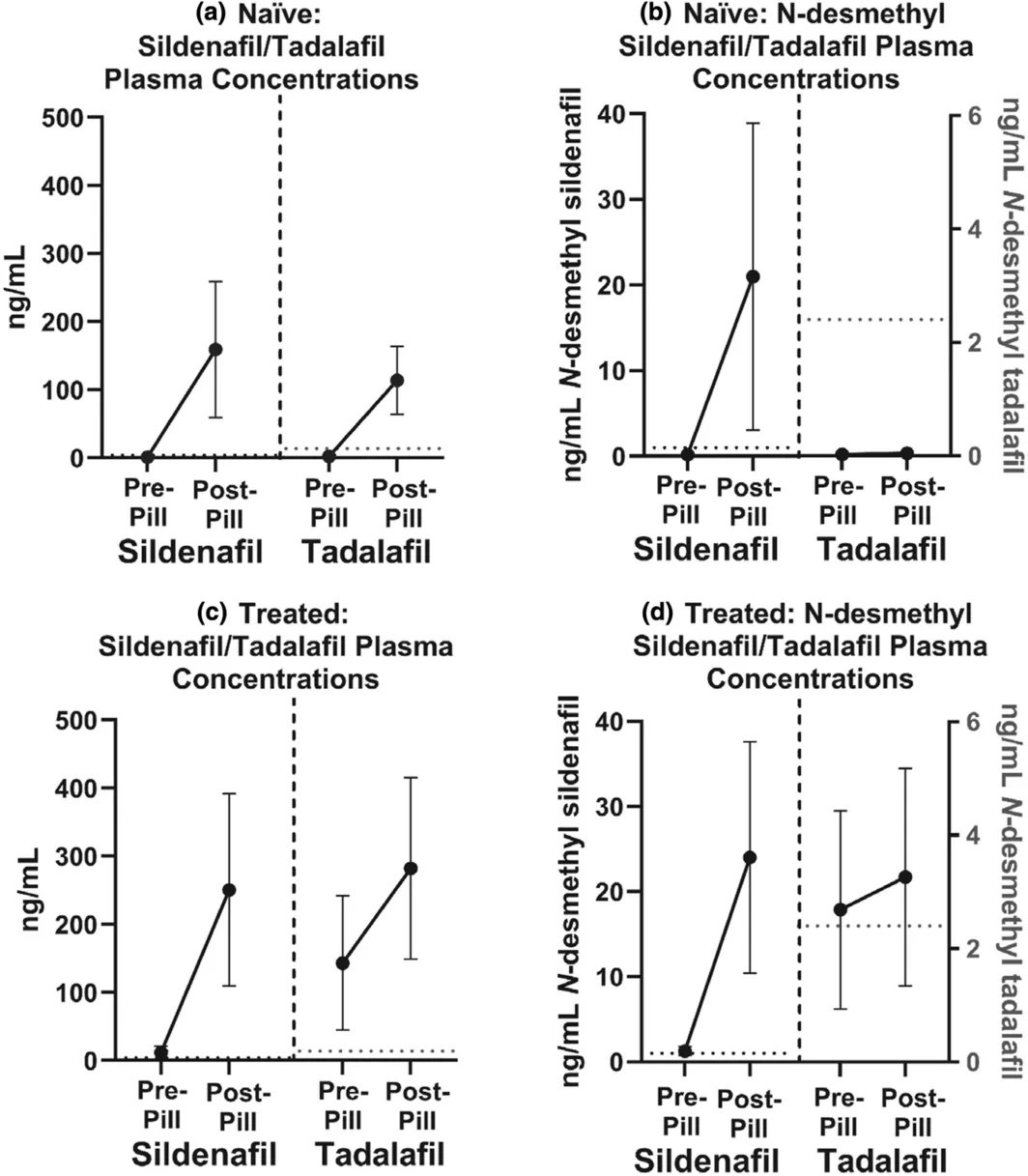
Mean (+ SD) pre‐pill and one‐hour post‐pill plasma concentrations of A/C: Sildenafil and tadalafil (ng/ml) and B/D: N‐desmethyl sildenafil and N‐desmethyl tadalafil (ng/ml) at treatment‐naïve (a, b) and treated (c, d) timepoints. Horizontal dotted lines indicate LLOQ limits (in black for sildenafil and N‐desmethyl sildenafil, and in gray for tadalafil and N‐desmethyl tadalafil). Arithmetic means were used.

Treatment‐naïve (*n* = 4): Pre‐pill measurements of plasma sildenafil and *N*‐desmethyl sildenafil were below LLOQ (Figure 3a,b, Tables S9 and S10). One‐hour post‐pill measurements ranged from 22 to 242 ng/mL (mean 159 ± 100 ng/mL) for sildenafil and below LLOQ (for 1 of 4 subjects) to 42 ng/mL (mean 21 ± 18 ng/mL) for *N*‐desmethyl sildenafil.

Treated (*n* = 8): Pre‐pill measurements ranged from below LLOQ (for 2 of 8 subjects) to 29 ng/mL (mean 12 ± 10 ng/mL) for sildenafil (Figure 3c,d, Tables S9 and S10). *N*‐desmethyl sildenafil measurements were below LLOQ for 6 of 8 subjects. One‐hour post‐pill measurements ranged from 91 to 506 ng/mL (mean 251 ± 141 ng/mL) for sildenafil and below LLOQ (for 1 of 8 subjects) to 39 ng/mL (mean 24 ± 14 ng/mL) for *N*‐desmethyl sildenafil.

#### 3.2.2 Tadalafil

Treatment‐naïve (*n* = 4): Pre‐pill measurements of plasma tadalafil and *N*‐desmethyl tadalafil were below LLOQ (Figure 3a,b, Tables S11 and S12). One‐hour post‐pill measurements ranged from 95 to 182 ng/mL (mean 114 ± 50 ng/mL) for tadalafil and remained below LLOQ for *N*‐desmethyl tadalafil.

Treated (*n* = 8): Pre‐pill measurements ranged from below LLOQ (for 1 of 8 subjects) to 302 ng/mL (mean 143 ± 99 ng/mL) for tadalafil (Figure 3c,d, Tables S11 and S12). *N*‐desmethyl tadalafil measurements ranged from below LLOQ (for 3 of 8 subjects) to 6 ng/mL (mean 2.7 ± 1.8 ng/mL). One‐hour post‐pill measurements ranged from 93 to 476 ng/mL (mean 282 ± 133 ng/mL) for tadalafil and below LLOQ (for 2 of 8 subjects) to 6 ng/mL (mean 3.3 ± 1.9 ng/mL) for *N*‐desmethyl tadalafil.

### 3.3 Plasma metabolomics

**FIGURE 4:**
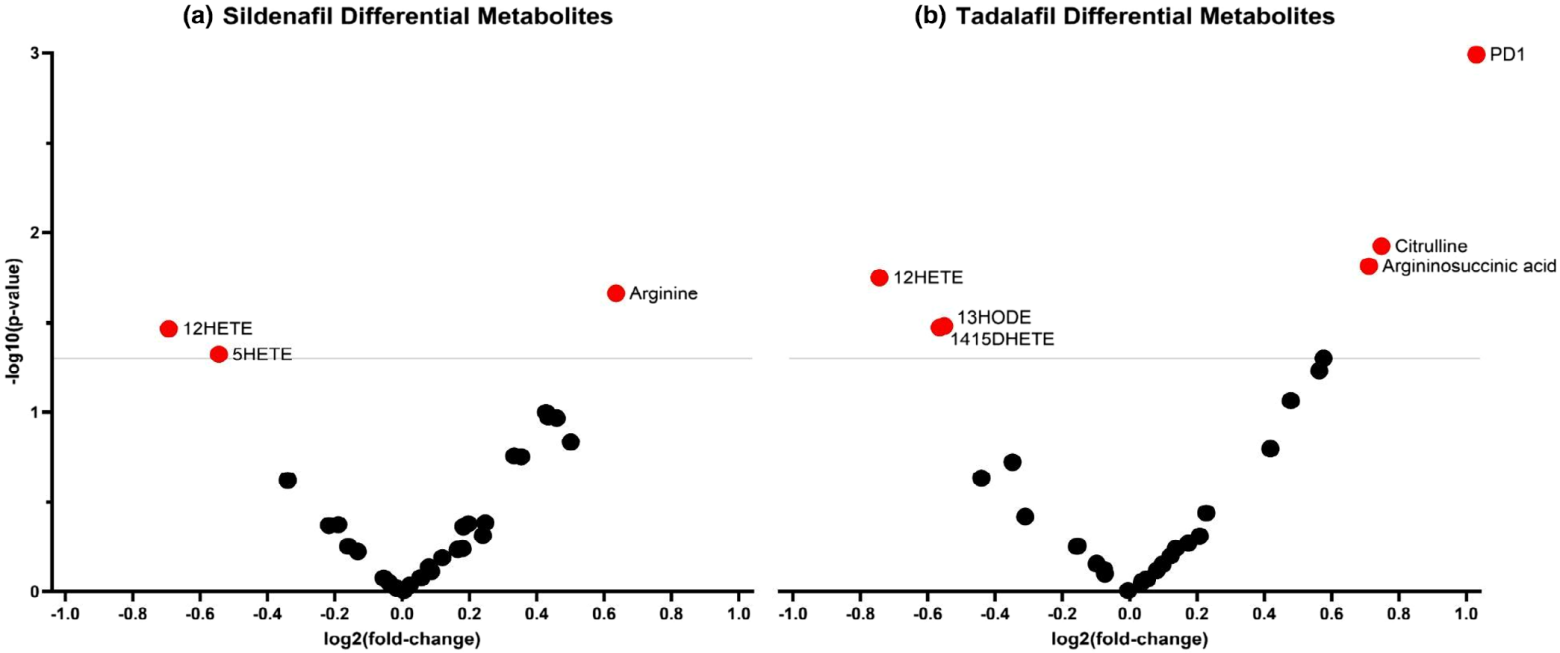
Volcano plots depicting significant differential metabolites (in red, *α* = 0.05 line in gray) between pre‐ and post‐treatment plasma samples in subjects that underwent 4 weeks of treatment with (a) sildenafil or (b) tadalafil. Positive log2(fold‐change) values indicate an increase in that metabolite following 4 weeks of treatment with PDE‐5 inhibitor and negative log2(fold‐change) values indicate a decrease in that metabolite following 4 weeks of treatment with PDE‐5 inhibitor. 12HETE: 12‐hydroxyeicosatetraenoic acid, 13HODE: 13‐hydroxyoctadecadienoic acid, 1415DHETE: 14,15‐dihydroxyeicosatrienoic acid, 5HETE: 5‐hydroxyeicosatetraenoic acid, PD1: Neuroprotectin D1.

Thirty‐two metabolites, including 27 lipid mediators and 5 arginine metabolites, were present in the final dataset. Two metabolites each were filtered out of the sildenafil and tadalafil datasets in Metaboanalyst, leaving 30 metabolites in each dataset for differential analyses (Table S1). Following 4 weeks of treatment with sildenafil (*n* = 8), L‐arginine increased 1.55‐fold (*p* = 0.022), 12‐hydroxyeicosatetraenoic acid (12HETE) decreased 1.62‐fold (*p* = 0.034), and 5‐hydroxyeicosatetraenoic acid (5HETE) decreased 1.46‐fold (*p* = 0.048), respectively, over 4 weeks of treatment (Figure 4a). Following 4 weeks of treatment with tadalafil (*n* = 8), protectin D1 (PD1) increased 2.04‐fold (*p* = 0.001), citrulline increased 1.68‐fold (*p* = 0.012), argininosuccinic acid (biologically argininosuccinate) increased 1.64‐fold (*p* = 0.015), 12HETE decreased 1.67‐fold (*p* = 0.018), 14,15‐dihydroxyeicosatrienoic acid (1415DHETE) decreased 1.48‐fold (*p* = 0.034), and 13‐hydroxyoctadecadienoic acid (13HODE) decreased 1.47‐fold (*p* = 0.033) over 4 weeks of treatment (Figure 4b).

### 3.4 Skeletal muscle transcriptomics

**FIGURE 5:**
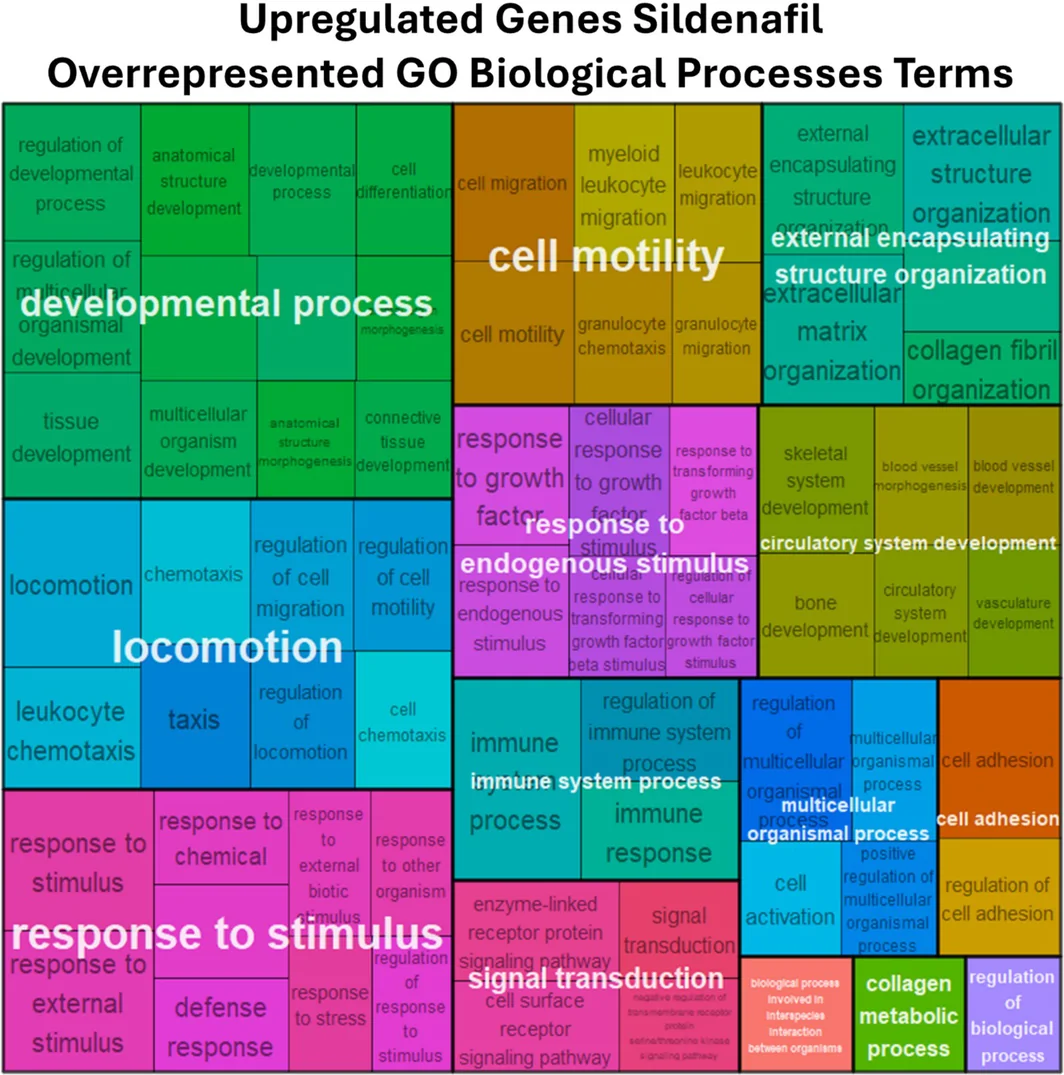
Treemap depicting enriched GO Biological Processes terms of upregulated genes in skeletal muscle following 4 weeks of sildenafil treatment. Cluster representatives are the terms with the largest number of genes in the cluster and are not necessarily of higher order than nested terms. Larger rectangle sizes indicate more significant terms. Font size is non‐informative.

There were no DEGs following 4 weeks of treatment with tadalafil (*n* = 7; there was insufficient tissue for analysis for one subject's post‐treatment biopsy), and thus no enriched GO terms. Following 4 weeks of treatment with sildenafil (*n* = 8), there were 23 genes that were downregulated (Table S2) and 101 genes that were upregulated (Table S3) following 4 weeks of treatment. There were no enriched GO terms of the downregulated genes. There were 68 GO Biological Processes terms that were enriched based on the upregulated genes. Enriched GO Biological Processes cluster representatives included cell adhesion, cell motility, developmental process, circulatory system development, external encapsulating structure organization, immune system process, multicellular organismal process, response to endogenous stimulus, and signal transduction (Figure 5). The three individual terms with the highest gene ratios were extracellular matrix disassembly (0.24), granulocyte chemotaxis (0.17), and collagen fibril organization (0.16, Figures S2 and S3). The term with the lowest p‐value was immune system process.

### 3.5 Skeletal muscle proteomics

Following 4 weeks of treatment with tadalafil (*n* = 7; there was insufficient tissue for analysis of one subject's post‐treatment biopsy), there were 116 proteins downregulated (Table S5) and 2 proteins upregulated (Table S6) following treatment. There were no GO Biological Components terms that were enriched based on the downregulated proteins.

**FIGURE 6:**
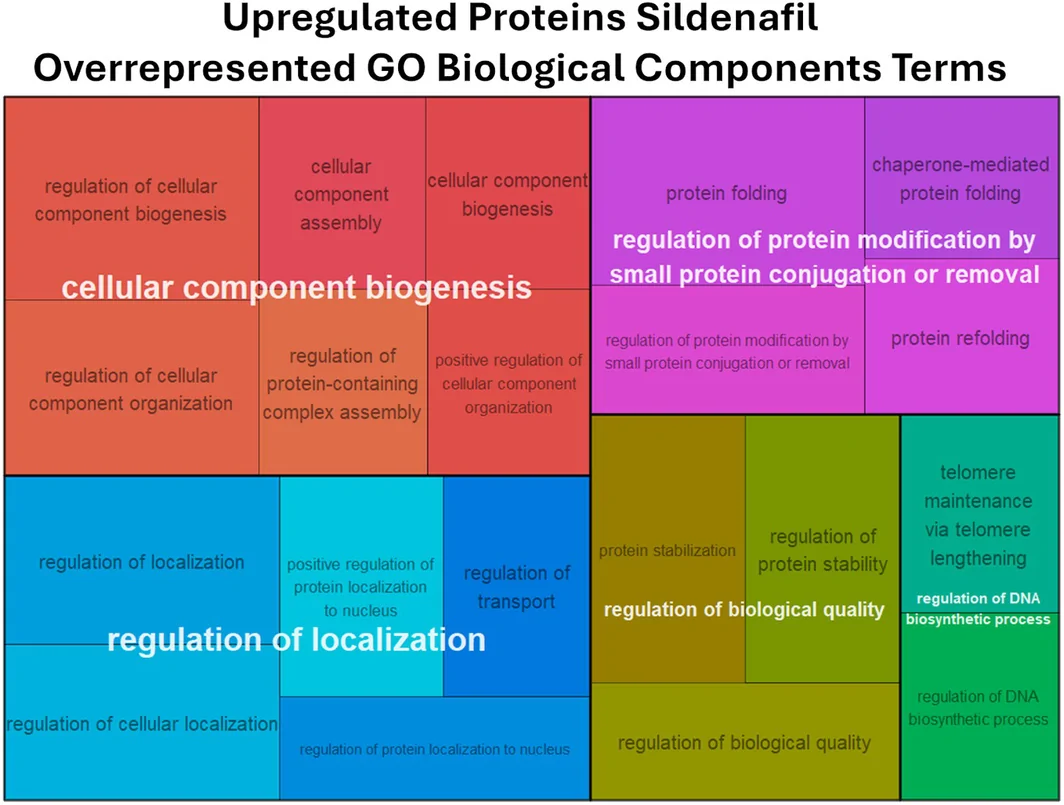
Treemap depicting enriched GO Biological Processes terms of upregulated proteins in skeletal muscle following 4 weeks of sildenafil treatment. Cluster representatives are the terms with the largest number of genes/proteins in the cluster and are not necessarily of higher order than nested terms. Larger rectangle sizes indicate more significant terms. Font size is non‐informative.

Following 4 weeks of treatment with sildenafil (*n* = 8), there were 10 proteins downregulated (Table S7) and 136 proteins upregulated (Table S8) following treatment. There were 20 GO Biological Processes terms that were enriched based on upregulated proteins (Figure 6). Enriched GO Biological Processes cluster representatives of upregulated proteins included cellular component biogenesis, regulation of localization, regulation of biological quality, regulation of protein modification by small protein conjugation or removal, and regulation of DNA biosynthetic process. The individual term with the highest fold enrichment was protein refolding (Figure S4).

There was some overlap in the transcriptomics and proteomics results for the sildenafil comparisons. Three genes were upregulated along with their corresponding proteins: peptidyl arginine deiminase 2 (*PADI2*), transforming growth factor beta induced (*TGFBI*), and coagulation factor XIII A chain (*F13A1*).

### 3.6 Multi‐omics integration

**FIGURE 7:**
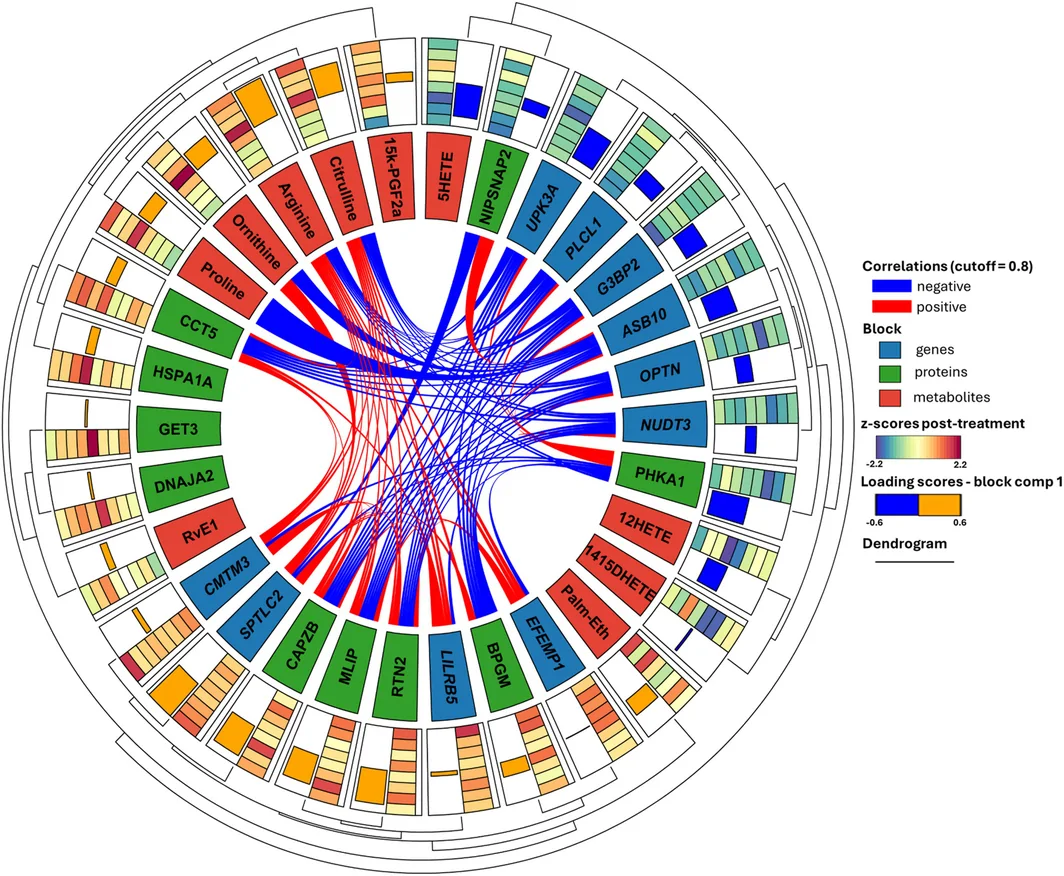
Visualization of DIABLO model results integrating key metabolomics, transcriptomics, and proteomics variables to discriminate post‐treatment from pre‐treatment samples following 4 weeks of treatment with sildenafil (*N* = 8). Tracks are described from innermost to outermost, as reflected by the legend. The first track is a chord diagram illustrating inter‐block correlations greater than an absolute value of 0.8 (chord width is non‐informative). The second track depicts variable identities and block memberships. On the third track, the heatmaps represent post‐treatment *z*‐scores calculated using extracted within‐subject variation (pre‐treatment *z*‐scores are the additive inverses of these values) and the bar plots demonstrate loading scores for the first (and only) component for each block. The fourth track is a dendrogram portraying hierarchical clustering results on variable *z*‐scores using Euclidean distance and complete linkage. 12HETE, 12‐hydroxyeicosatetraenoic acid; 1415DHETE, 14,15‐dihydroxyeicosatrienoic acid; 15k‐PGF2a, 15‐keto prostaglandin F2α; 5HETE, 5‐hydroxyeicosatetraenoic acid; ASB10, ankyrin repeat and SOCS box containing 10; BPGM, bisphosphoglycerate mutase; CAPZB, capping actin protein of muscle Z‐line subunit beta; CCT5, chaperonin containing T‐complex protein 1 subunit 5; CMTM3, CKLF like MARVEL transmembrane domain containing 3; DNAJA2, DNAJ heat shock protein family (Hsp40) member A2; EFEMP1, EGF containing fibulin extracellular matrix protein 1; G3BP2, GTPase‐activating protein SH3 domain‐binding protein stress granule assembly factor 2; GET3, guided entry of tail‐anchored proteins factor 3; ATPase, HSPA1A: heat shock protein family A (Hsp70) member 1A; LILRB5, leukocyte immunoglobulin like receptor B5; MLIP, muscular LMNA interacting protein; NIPSNAP2, nipsnap homolog 2; NUDT3, nudix hydrolase 3; OPTN, optineurin; Palm‐Eth, palmitoylethanolamide; PHKA1, phosphorylase kinase regulatory subunit alpha 1; PLCL1, phospholipase C like 1; RTN2, reticulon 2; RvE1, resolvin E1; SPTLC2, serine palmitoyltransferase long chain base subunit 2; UPK3A, uroplakin 3A.

As specified in the methods section, the DIABLO models selected 10 variables for the component for each block, or ‘omics data set, to best discriminate between the pre‐ and post‐treatment timepoints. Only interblock correlations greater than an absolute value of 0.8 are reported. For the sildenafil group (*n* = 8), 8/10 genes, 10/10 proteins, and 3/10 metabolites selected were also differentially expressed (Figure 7). The variable that had the most influence on the gene block component was serine palmitoyltransferase long chain base subunit 2 (*SPTLC2*), which increased following treatment and was positively coexpressed with 5 proteins and 3 metabolites. It was also negatively correlated with 2 proteins. The variable with the most influence on the protein block component was phosphorylase kinase regulatory subunit alpha 1 (PHKA1), which decreased following treatment and was positively correlated with 6 genes and negatively correlated with 4 genes and 2 metabolites. The variable contributing the highest loading to the metabolomic block component was L‐arginine, which was increased following treatment and positively coexpressed with 4 genes and 5 proteins and negatively correlated with 6 genes and 1 protein.

**FIGURE 8:**
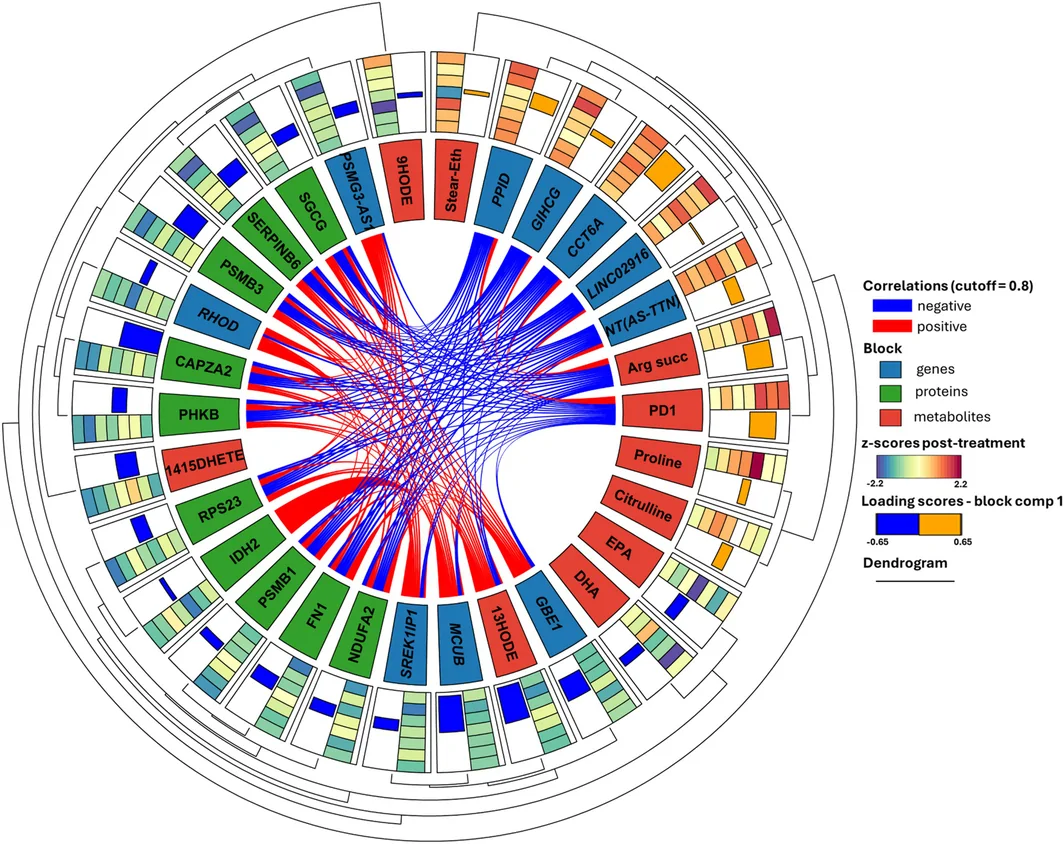
Visualization of DIABLO model results integrating key metabolomics, transcriptomics, and proteomics variables to discriminate post‐treatment from pre‐treatment samples following 4 weeks of treatment with tadalafil (*N* = 7). Tracks are described from innermost to outermost, as reflected by the legend. The first track is a chord diagram illustrating inter‐block correlations greater than an absolute value of 0.8 (chord width is non‐informative). The second track depicts variable identities and block memberships. On the third track, the heatmaps represent post‐treatment *z*‐scores calculated using extracted within‐subject variation (pre‐treatment *z*‐scores are the additive inverses of these values) and the bar plots demonstrate loading scores for the first (and only) component for each block. The fourth track is a dendrogram portraying hierarchical clustering results on variable *z*‐scores using Euclidean distance and complete linkage. 13HODE, 13‐hydroxyoctadecadienoic acid; 1415DHETE, 14,15‐dihydroxyeicosatrienoic acid; 9HODE, 9‐hydroxyoctadecadienoic acid; Arg succ, argininosuccinic acid; CAPZA2, capping actin protein of muscle Z‐line subunit alpha 2; CCT6A, chaperonin containing TCP1 subunit 6A; DHA, docosahexaenoic acid; EPA, eicosapentaenoic acid; FN1, fibronectin 1; GBE1, 1,4‐alpha‐glucan branching enzyme 1; GIHCG, long noncoding RNA gradually increased during hepatocarcinogenesis/inhibitor of miR‐200b/200a/429 expression; IDH2, isocitrate dehydrogenase (NADP(+)) 2; LINC02916, long intergenic non‐protein coding RNA 2916; MCUB, mitochondrial calcium uniporter dominant negative subunit beta; NDUFA2, NADH:ubiquinone oxidoreductase subunit A2; NT(AS‐TTN), novel transcript, antisense to titin (ENSG00000279598); PD1, protectin D1; PHKB, phosphorylase kinase regulatory subunit beta; PPID, peptidylprolyl isomerase D; PSMB1, proteasome 20S subunit beta 1; PSMB3, proteasome 20S subunit beta 3; PSMG3‐AS1, proteasome assembly chaperone 3 antisense RNA 1 (head to head); RHOD, ras homolog family member D; RPS23, ribosomal protein S23; SERPINB6, serpin family B member 6; SGCG, sarcoglycan gamma; SREK1IP1, splicing regulatory glutamine/lysine‐rich protein 1–interacting protein 1; Stear‐Eth, stearoylethanolamide.

For the tadalafil group (*n* = 7), 5/10 metabolites and 9/10 proteins selected were also differentially expressed (Figure 8). The variable with the most influence on the gene block component was mitochondrial calcium uniporter dominant negative subunit beta (*MCUB*), which was decreased following treatment and positively correlated with all proteins and 1 metabolite, as well as negatively correlated with 2 metabolites. The variable with the most influence on the protein block component was capping actin protein of muscle Z‐line subunit alpha 2 (CAPZA2), which decreased following treatment and was positively correlated with 5 genes and 1 metabolite and negatively correlated with 5 genes and 2 metabolites. The variable with the highest loading on the metabolite block component was 13‐hydroxyoctadecadienoic acid (13‐HODE), which decreased following treatment and was positively correlated with 4 genes and 8 proteins and negatively correlated with 3 genes.

### 3.7 Skeletal muscle immunohistochemistry

**FIGURE 9:**
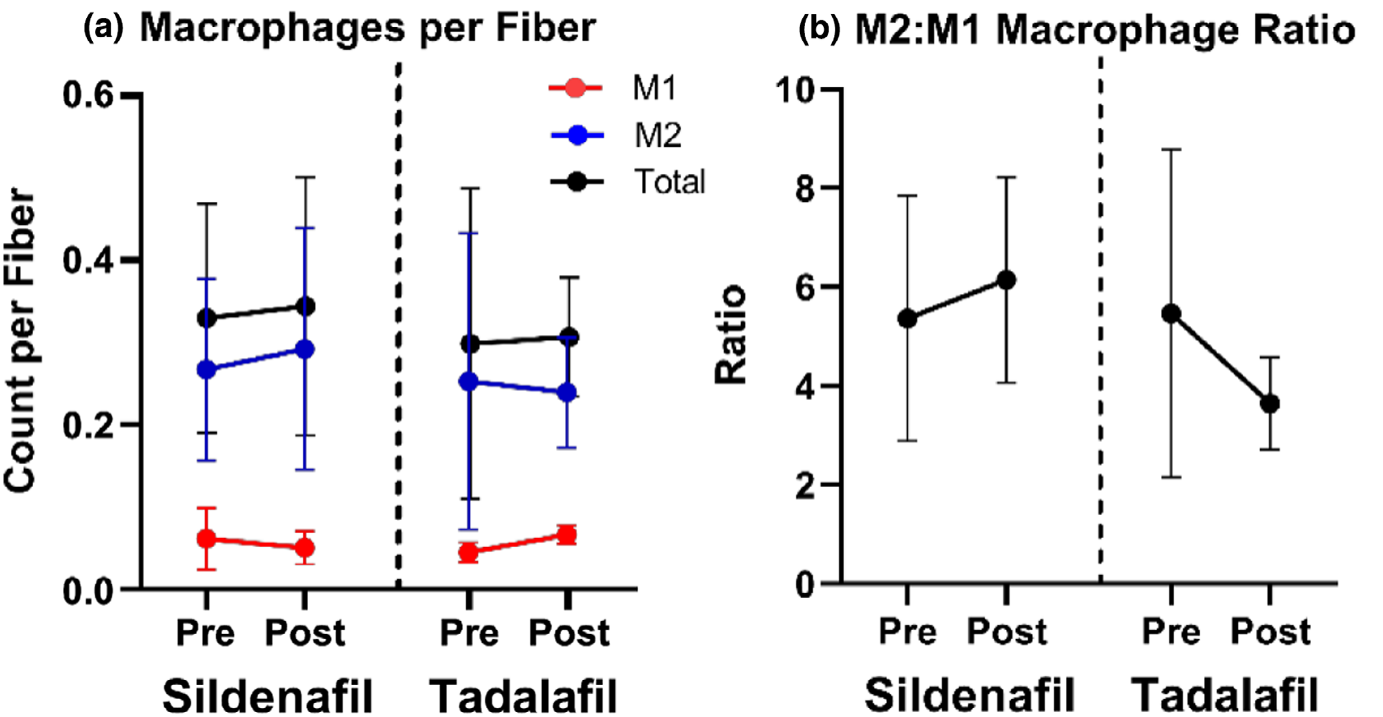
Skeletal muscle (a) macrophage counts per fiber and (b) ratios (mean + SD) prior to and following 4 weeks of treatment with sildenafil (*n* = 6) or tadalafil (*n* = 6).

Based on the decrease observed in serum proinflammatory metabolites following PDE‐5 inhibitor treatment, we decided to quantify macrophage subtypes in the skeletal muscle samples to determine whether there was a similar anti‐inflammatory signal. Two subjects from the sildenafil group and two subjects from the tadalafil group did not have paired pre‐post samples due to insufficient or damaged tissue from one or both timepoints. Paired *t*‐tests did not indicate significant changes in M1, M2, or total macrophage counts per fiber or M2:M1 ratio following 4 weeks of sildenafil treatment (*n* = 6, Figure 9). Following 4 weeks of tadalafil treatment (*n* = 6), uncorrected paired *t*‐tests indicated an increase in M1 macrophages, which was non‐significant after correcting for multiple testing (raw *p* value 0.001, *q* value 0.127). As mentioned previously, mixed effects models were also conducted for these data, as there were missing data and it appeared that macrophage counts increased during placebo (Figure S13). Mixed effects models comparing week 4 and week 8 measurements, taking week 0 measurements, treatment sequence, treatment, and period into account, did not indicate significant changes in total, M1, or M2 macrophage counts per fiber or M2:M1 ratio with PDE‐5 inhibitor treatment (Table S4, *p* = 0.063). Treatment sequence and period effects were not significant for any of the models.

## 4 DISCUSSION

In this study of responses to PDE‐5 inhibition, the results suggested responses common to both sildenafil and tadalafil as well as divergent responses to the two PDE‐5 inhibitors. Both tadalafil and sildenafil were associated with reduced serum eicosanoids and increased pathway components for arginine biosynthesis. Tadalafil had limited effects on the skeletal muscle proteome and transcriptome, while sildenafil upregulated gene ontology terms related to immunity and protective effects for bone, DNA integrity, and skeletal muscle function. These results were not reinforced by significant improvements in functional strength or endurance or in measures of perceptual fatigue in either the sildenafil or the tadalafil group, perhaps due to limited sample sizes and treatment durations.

### 4.1 Pharmacokinetics

Average 1‐h post‐pill concentrations of sildenafil at treatment‐naïve and treated timepoints were 159 ± 100 and 251 ± 141 ng/mL, respectively. These values are generally in range with previously reported geometric mean peak plasma concentrations ranging from 159 to 271 ng/mL within 0.8–1.5 h following a 50 mg dose in healthy men (Muirhead et al., 2002; Nichols et al., 2002). The treatment‐naïve post‐pill measurement for one individual was lower than expected at 22 ng/mL. This individual had a post‐pill concentration >400 ng/mL during the treated timepoint; it is unclear why their response was so different among weeks. Another subject had a post‐pill concentration of >500 ng/mL during the treated timepoint; the other six subjects treated with sildenafil had post‐pill concentrations of 225 ng/mL or less during the treated timepoint. The two subjects with the highest post‐pill concentrations at the treated timepoint were females. Plasma concentrations of sildenafil can vary in relation to genetic variants linked to hepatic first‐pass metabolism (de Denus et al., 2018) and activity of CYP3A4, the liver enzyme responsible for metabolizing sildenafil, is known to decrease during menopause (Sommer et al., 2025). We did not collect information on CYP3A4 phenotype or menopausal status in this study, so we are unable to investigate whether estrogen availability could be a contributing factor.

Average one‐hour post‐pill concentrations of *N*‐desmethyl sildenafil at treatment‐naïve and treated timepoints were 21 ± 18 and 24 ± 14 ng/mL, respectively. These values are lower than previously reported geometric mean peak plasma concentrations ranging from ~70 to 126 ng/mL within 0.8–1 h following a 50 mg dose (Muirhead et al., 2002; Nichols et al., 2002). *N*‐desmethyl sildenafil is known to be an active metabolite, with ~50% potency relative to sildenafil and accounting for ~20% of sildenafil's effects (Kouvelas et al., 2009; Mehrotra et al., 2007).

Average 1‐h post‐pill concentrations of tadalafil at treatment‐naïve and treated timepoints were 114 ± 50 and 282 ± 133 ng/mL, respectively. The mean concentration at treatment‐naïve timepoints is slightly lower than previously reported geometric mean peak concentrations of ~180–193 ng/mL following a 10 mg dose in healthy men, but those maximum concentrations were measured over a longer duration and occurred between 1 and 2.5 h following dose administration (Forgue et al., 2007). The mean concentration at treated timepoints exceeded the previously reported geometric mean peak concentrations, but fell within range of median values predicted by a simulated model of peak tadalafil concentration in men with erectile dysfunction, in which 90% of values fell between ~80 and 280 ng/mL (median ~ 150 mg/mL) (Trocóniz et al., 2007). The higher concentrations seen in this study are likely due to the longer half‐life of tadalafil and subsequent longer duration of action compared to sildenafil (24–36 h vs. 4–8 h), resulting in accumulation of the drug (Kouvelas et al., 2009; Mehrotra et al., 2007). Mean pre‐pill concentration of tadalafil during treated timepoints was 143 ± 99 ng/mL, slightly more than arithmetic mean concentrations of ~70–100 ng/mL seen ~24 h following a 10 mg dose in healthy men (Forgue et al., 2007).

No previously reported values for *N*‐desmethyl tadalafil were found in the literature against which to make a comparison for this study. Many of the reported concentrations were below the LLOQ, but a general increase in concentrations during active treatment with the drug can be seen, as well as an absence of signal during placebo treatment.

### 4.2 Plasma metabolomics

In this study, plasma concentrations of eicosanoids typically associated with inflammation were reduced following 4 weeks of PDE‐5 inhibitor treatment. Eicosanoids are bioactive lipid signaling molecules derived from the metabolism of cellular 20‐carbon polyunsaturated fatty acids (PUFAs), primarily arachidonic acid, by enzymes including lipoxygenases and cytochrome P450 (Moore & Pidgeon, 2017). 12‐HETE, the main metabolite of 12‐lipoxygenase (12‐LOX), decreased following both sildenafil and tadalafil treatment. 12‐HETE and/or 12‐LOX have been linked to oxidative stress, endothelial dysfunction, neuroinflammation, carcinogenesis, and cancer metastasis (Chen & Zou, 2022a; Honn et al., 1994; Kulkarni et al., 2021; Nie et al., 1998; Othman et al., 2013; Yoshimura et al., 2004; Zarbock et al., 2009). Previous research showed that 12‐LOX activity in platelets was inhibited by administration of L‐arginine or sodium nitroprusside, which generate NO (Fujimoto et al., 1998; Nakatsuka & Osawa, 1994) cGMP signaling that is prolonged by PDE‐5 inhibitors. 5‐HETE, the main metabolite of 5‐LOX, decreased following sildenafil treatment. Previous research found that drugs releasing NO intracellularly inhibited 5‐LOX via direct nitrosylation (Roos et al., 2018). 5‐HETE and/or 5‐LOX have been implicated in tumorigenesis and cancer progression, as well as mild cognitive impairment and Alzheimer's disease (Ebright et al., 2022; Fang et al., 2017; Faronato et al., 2007; Greene et al., 2011; Ikonomovic et al., 2008; Matsuyama et al., 2005; Moore & Pidgeon, 2017; Steinhilber et al., 2010). 5‐LOX and 12‐LOX are additionally both implicated in microglia‐mediated neuroinflammation (Chen & Zou, 2022b). 13‐HODE and 14,15‐DiHETrE, which decreased following tadalafil treatment, have both been associated with cognitive impairment (Kotlęga et al., 2021; Nelson et al., 2014). It is not clear by what mechanism these inflammatory eicosanoids were reduced with PDE‐5 inhibitor treatment, but it is possible that 12‐LOX and 5‐LOX activity was inhibited, possibly via nitrosylation. Plasma concentrations of protectin D1 (PD1—a docosanoid, which are lipid mediators originating from 22‐carbon PUFAs), which is derived from docosahexaenoic acid, were increased following treatment with tadalafil. PD1 is known to have anti‐inflammatory and anti‐oxidative properties and can be biosynthesized by macrophages at tissue injury sites (Bazan, 2005; Hong et al., 2014; Zhou et al., 2020).

Prior studies found that low‐grade inflammation is associated with self‐reported measures of increased fatigue and mood disturbance (Milaneschi et al., 2021; Montoya et al., 2017). In this study, we did not detect a significant effect of 4 weeks of PDE‐5 inhibitor treatment on self‐reported measures of fatigue and mood (Figures S11 and S12) despite the significant reduction in inflammatory eicosanoids and increase in anti‐inflammatory docosanoids. However, these are only a small portion of the plasma biomarkers associated with inflammation, and participants in this study were from a presumed healthy population.

**FIGURE 10:**
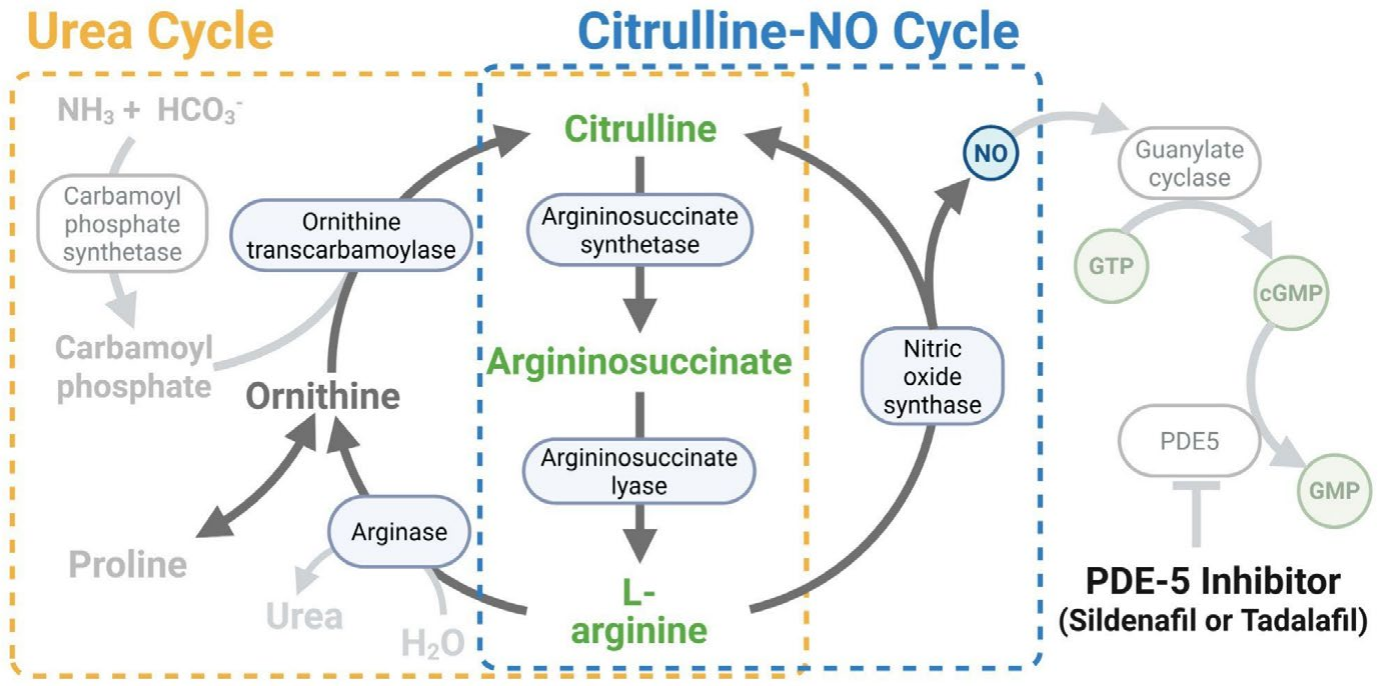
L‐arginine biosynthesis related pathways. Metabolites increased following sildenafil and/or tadalafil treatment are in green. Created with BioRender.

Four weeks of PDE‐5 inhibitor treatment appears to upregulate arginine biosynthesis pathways (Figure 10). KEGG pathway analysis of colorectal cancer cells treated with tadalafil in vitro similarly revealed enrichment of the arginine biosynthesis pathway (Zhao et al., 2022). Two amino acids, argininosuccinate and citrulline, were increased following tadalafil treatment. L‐arginine, argininosuccinic acid, and citrulline are all intermediates of both the urea cycle and the citrulline‐NO cycle. Most endogenous L‐arginine synthesis in mammals occurs via the intestinal‐renal axis in the absence of a full urea cycle. Citrulline, synthesized from glutamine and potentially proline by small intestinal enterocytes, is converted in the kidneys to argininosuccinate via argininosuccinate synthetase and then to arginine via argininosuccinate lyase (Brosnan & Brosnan, 2004; Peranzoni et al., 2007). These two urea cycle enzymes, in conjunction with nitric oxide synthase (NOS), are also responsible for the citrulline‐NO cycle, in which citrulline is converted first to argininosuccinate and then to arginine, which is converted to NO and citrulline by NOS. This cycle occurs in several cell types, including neuronal cells, endothelial cells, and macrophages (Flam et al., 2007).

PDE‐5 inhibition alone doesn't explain the increase of arginine and other arginine biosynthesis pathway intermediates. One explanation could be that treatment with PDE‐5 inhibitors downregulated arginase, as has been demonstrated in studies of murine myeloid‐derived suppressor cells (Serafini et al., 2006). Arginase is an enzyme of the urea cycle which competes with NOS for L‐arginine as a substrate (Durante et al., 2007). Reduced arginase expression or activity could result in larger amounts of serum arginine available for use by NOS. Another explanation could be that PDE‐5 inhibitor treatment downregulated inducible NOS (iNOS), as has been observed in some preclinical studies (Garcia et al., 2014; Serafini et al., 2006; Zhao et al., 2011). iNOS is associated with inflammation and has been shown to have detrimental effects, unlike endothelial NOS (eNOS), which is protective of the endothelium (Ding et al., 2014) and shows increased activity or expression following treatment with PDE‐5 inhibitors (Gebska et al., 2011; Kim & Lee, 2020; Shue et al., 2014). Yet another explanation could be an increase in citrulline synthesis. Blood citrulline concentrations are reduced in patients with impaired gut barriers and intestinal enteropathies such as Crohn's disease and celiac disease, thought to be caused primarily by reduced mass and/or functioning of enterocytes (Crenn et al., 2008; Fragkos & Forbes, 2018). PDE‐5 inhibitors protect against injury in both the small intestine and the colon (El‐Tanbouly & Abdelrahman, 2022; Hunter et al., 2022), so it is possible that the function of small intestinal enterocytes was improved following 4 weeks of treatment with PDE‐5 inhibitor.

### 4.3 Skeletal muscle transcriptomics

In this study, there were a number of DEGs in the skeletal muscle of subjects treated with sildenafil but not subjects treated with tadalafil. This is surprising, as sildenafil is active for a much shorter duration than tadalafil (4–8 h compared to 24–36 h) (Mehrotra et al., 2007). Many GO terms associated with genes upregulated following sildenafil treatment are similar to those enriched following long‐term aerobic training, including extracellular matrix organization, angiogenesis, cell adhesion, leukocyte migration, and response to stimulus (Makhnovskii et al., 2021). Some of the terms were also similar to those enriched following resistance training‐induced hypertrophy (Lim et al., 2022) and muscle regrowth (Chaillou et al., 2015), which aligns with our previous study which observed an increase in skeletal muscle protein synthesis following 8 days of treatment with sildenafil (Sheffield‐Moore et al., 2013).

Genes related to immune response were upregulated following sildenafil treatment. Preclinical and clinical studies indicate that PDE‐5 inhibitors have immunomodulatory properties and promote antitumor immunity in cancer (Califano et al., 2015; Morsi et al., 2023; Tang et al., 2025; Weed et al., 2015). Genes related to cell motility, migration, and chemotaxis, in particular for leukocytes and granulocytes, were also upregulated. Migration of leukocytes is often in response to inflammation and tissue injury (Choi, 2009). The upregulation of genes related to cell adhesion could also be indicative of inflammation (Ma et al., 2023). Because biopsies at each study visit were taken in close proximity to previous biopsy sites, it is possible that tissue injury from the previous biopsy resulted in local inflammation and recruitment of immune cells to the site.

Genes related to blood vessel and bone development were upregulated following sildenafil treatment. In vitro and in vivo preclinical experiments concluded that sildenafil stimulates angiogenesis through a protein kinase G‐dependent pathway independent of NO signaling (Pyriochou et al., 2007; Senthilkumar et al., 2007). Preclinical studies also indicated that PDE‐5 inhibitors are osteoprotective and accelerate fracture healing (Histing et al., 2011; Hu et al., 2025; Kim et al., 2020; Menger et al., 2023; Pal et al., 2020), although these effects may not extend to aged models or in the case of high dosages (Gong et al., 2014; Menger et al., 2024).

### 4.4 Skeletal muscle proteomics

The majority of the differentially expressed proteins following sildenafil treatment were upregulated rather than downregulated, similar to the DEGs following treatment with sildenafil. Results from another study investigating the effect of sildenafil on the skeletal muscle proteome showed a similar pattern, with >93% of differentially expressed proteins being upregulated (Wu et al., 2023). Proteins related to protein folding and refolding of misfolded proteins, important components of the protein quality control system (Bozaykut et al., 2014), were upregulated following treatment with sildenafil. This indicates that the unfolded protein response, a cellular stress response, may have been activated following sildenafil treatment. Although the unfolded protein response is sometimes associated with disease (Hetz, 2012), it also appears to play an important role in skeletal muscle adaptation to exercise with activation posited to improve skeletal muscle function and metabolism (Hart et al., 2019; Wu et al., 2011). In addition, an increase in protein synthesis could result in activation of the unfolded protein response if the rate at which proteins are being produced exceeds the rate at which they can be folded in the endoplasmic reticulum (Hussain et al., 2014; Ogborn et al., 2014).

Proteins related to telomere maintenance via lengthening and DNA biosynthetic process were upregulated following sildenafil treatment. Telomeres function as caps on the terminal ends of chromosomes, protecting DNA integrity. Telomere length tends to decrease with aging and in skeletal muscle cells is positively associated with exercise level and inversely related to age‐related disorders such as cancer, chronic pain, and cardiovascular disease (Arsenis et al., 2017).

Although a large number of proteins were downregulated following tadalafil treatment, very few GO terms were enriched, suggesting that the proteins were not involved in similar functions or pathways. This contrasted with the large number of enriched GO terms associated with upregulated proteins following sildenafil treatment. This is similar to the transcriptomics data in that many genes were upregulated following sildenafil treatment, but no genes were differentially expressed following tadalafil treatment. As previously mentioned, this is unexpected, as tadalafil is active for much longer than sildenafil. This brings into question whether it is possible that some of the effects are off‐target. Sildenafil and tadalafil are structurally dissimilar and inhibit other phosphodiesterase isozymes to varying degrees. Sildenafil cross‐reacts with PDE‐1 and PDE‐6 to a higher degree than tadalafil, and tadalafil cross‐reacts with PDE‐11 to a greater degree than sildenafil. PDE‐1 is expressed in the brain, myocardium, and vascular smooth muscle, and inhibition can cause vasodilation, tachycardia, and likely decreased proliferation of vascular smooth muscle cells. PDE‐6 is expressed primarily in the retinal tissue, and inhibition can result in blurred vision. PDE‐11 is expressed in the testes, skeletal muscle, myocardium, liver, kidney, and pituitary, but little is known about its physiological role (and thus, effects of its inhibition) (Bischoff, 2004).

### 4.5 Multi‐omics integration

Strong coexpression patterns were observed in the DIABLO model multi‐omics results for both the sildenafil and tadalafil treatment groups. For the sildenafil data set, *SPTLC2*, which increased following treatment, was the most informative variable for the gene block component. *SPTLC2* is a protein‐coding gene for a subunit of serine palmitoyltransferase, which is responsible for the first stage of sphingolipid biosynthesis (Lone et al., 2020). Increased expression of *SPTLC2* in skeletal muscle is associated with aging (Laurila et al., 2022), but was also upregulated in *vastus lateralis* biopsies following an 8‐week aerobic training intervention in sedentary participants at risk for Type II diabetes (Goj, 2023). The variable with the highest loading score on the protein block component was PHKA1, which decreased following treatment. PHKA1 is a regulatory enzyme subunit of phosphorylase kinase (Francke et al., 1989), which is responsible for glycogen breakdown through the phosphorylation of glycogen phosphorylase (Robison & Colbran, 2013). PDE‐5 inhibition has been shown to enhance skeletal muscle glucose uptake from circulation (Ayala et al., 2007; Jansson et al., 2010), which could result in reduced reliance on muscle glycogen stores. The variable with the most impact on the metabolomic block component was L‐arginine, which increased following treatment and was discussed at length in the metabolomics section.

For the tadalafil data set, the most informative variable for the gene block component was *MCUB*, which decreased following treatment. The MCUB protein forms heteromers with and negatively regulates the mitochondrial calcium uniporter (MCU) protein, which is the pore‐forming subunit of the mitochondria calcium uniporter complex (mtCU), influencing calcium permeation across the channel (Checchetto & Szabò, 2019; Raffaello et al., 2013; Tarasova et al., 2019). While MCU overexpression increases mitochondrial calcium uptake and results in skeletal muscle hypertrophy, MCU silencing results in reduction of skeletal muscle fiber size in mice (Mammucari et al., 2015), so it is likely that a decrease in the MCUB, which would increase calcium uptake, would also induce skeletal muscle hypertrophy. Mitochondrial calcium uptake is reduced with aging and sarcopenia through downregulation of mitochondrial calcium uniporter regulator 1 (MCUR1) (Gherardi et al., 2025), so downregulation of MCUB could present as a potential intervention to augment calcium uptake and ameliorate muscle aging. However, the benefits of increased calcium uptake occur within a narrow window, as excessive uptake can result in overload and cellular dysfunction (Zhou et al., 2016). The variable with the highest loading for the protein block component was CAPZA2, which decreased following treatment. CAPZA2 is a subunit of capping actin protein of muscle Z‐line (CAPZ), which caps the barbed ends of actin filaments, the primary constituents of the actin cytoskeleton (Svitkina, 2018). PDE‐5 inhibitors increase phosphorylation of vasodilator‐stimulated phosphoprotein (VASP) (Yang et al., 2019), which interacts with actin filament ends to block capping proteins (Bear et al., 2002). The most influential variable for the metabolite block component was 13‐HODE, which decreased following treatment, and was discussed in the metabolomics section.

### 4.6 Skeletal muscle immunohistochemistry

In this study, there was no significant change in M1 or M2 macrophages following 4 weeks of sildenafil or tadalafil treatment. M1 macrophages are the more pro‐inflammatory phenotype and produce cytokines such as interleukin‐6 and tumor necrosis factor, while M2 macrophages are the more anti‐inflammatory or resolving phenotype responsible for tissue repair (Yunna et al., 2020). Prior studies indicate that PDE‐5 inhibitors can regulate inflammatory response in diverse tissues. Sildenafil shifted circulating renal and cardiac TIE2‐expressing monocytes from M1‐like to M2‐like polarization in a mouse model of diabetes, promoted an M2 phenotype in a mouse model of multiple sclerosis, and decreased macrophage infiltration in rat models of pulmonary hypertension and focal brain injury (Díaz‐Lucena et al., 2018; Kiss et al., 2014; Pifarré et al., 2010; Venneri et al., 2019). Sildenafil was shown in a mouse model of neonatal stroke to reduce overall macrophages and microglia and shift polarization toward an M2 phenotype, to ameliorate pro‐inflammatory responses induced by lipopolysaccharide exposure in N9 microglial cells, and to reduce microglial activation in the cerebellum of rats with hepatic encephalopathy (Moretti et al., 2016; Zhao et al., 2011).

## 5 LIMITATIONS

The main limitation for this study was the number of subjects. It is possible that more participants may have increased the effect size for some of our comparisons, specifically for the metabolomics and immunohistochemical datasets. In addition, repeat skeletal muscle biopsies conducted in close proximity may have resulted in local inflammation during later timepoints, potentially skewing the transcriptomic, proteomic, and immunohistochemical results.

## 6 CONCLUSIONS

Serum proinflammatory lipid signaling molecules decreased and arginine biosynthesis components increased following 4 weeks of treatment with sildenafil or tadalafil. In the skeletal muscle following sildenafil treatment, genes related to the immune response and bone and blood vessel development and proteins related to protein folding and telomere maintenance were increased, while there was no cohesive transcriptomic or proteomic signal in the tadalafil group. No significant differences in skeletal muscle macrophage subtypes or total macrophages were observed following treatment in either group. These results provide some evidence that PDE‐5 inhibitors have systemic anti‐inflammatory effects, but this was not corroborated locally in the skeletal muscle, possibly due to inflammation induced by proximity of pre‐ and post‐treatment biopsy sites. Future research should investigate effects of PDE‐5 inhibitors on local inflammation in various tissues, with special care taken to avoid undue interference caused by sampling techniques.

## AUTHOR CONTRIBUTIONS

**Kristen A. McGovern:** Data curation; formal analysis; methodology; software; validation; visualization. **Traver J. Wright:** Methodology; supervision; visualization. **Erik D. Marchant:** Data curation; investigation; methodology; resources; software; validation. **Sean P. Kilroe:** Data curation; investigation; methodology; resources; software; validation. **William J. Durham:** Conceptualization; data curation; investigation; methodology; project administration; resources; software; supervision; validation. **E. Lichar Dillon:** Conceptualization; data curation; investigation; methodology; project administration; resources; software; supervision; validation. **Marc M. Baum:** Data curation; investigation; methodology; resources; software; validation. **Michael P. Kinsky:** Investigation; project administration; resources; supervision. **William K. Russell:** Data curation; investigation; methodology; resources; software; validation. **Christopher S. Fry:** Data curation; investigation; methodology; resources; software; validation. **Pratik Gongloor:** Data curation; validation. **Richard B. Pyles:** Data curation; investigation; methodology; resources; software; validation. **Randall J. Urban:** Conceptualization; project administration; resources; supervision. **Blake B. Rasmussen:** Data curation; investigation; methodology; resources; software; supervision; validation. **Melinda Sheffield‐Moore:** Conceptualization; funding acquisition; investigation; methodology; project administration; resources; supervision.

## FUNDING INFORMATION

This study was funded by the Moody Endowment (M. Sheffield‐Moore). K. McGovern was supported by the NIH/NCATS UTMB TL1 Institutional Training Core (TL1TR001440) during the analyses and writing of the manuscript.

## CONFLICT OF INTEREST STATEMENT

Melinda Sheffield‐Moore and Marc Baum are co‐founders of Impervius Medical and have a pending patent application for a device that dispenses one of the drugs studied in this manuscript. This company was not formed when the study was conceived or completed and the device had not yet been invented. The remaining authors declare no conflicts of interest.

## ETHICS STATEMENT

This study was conducted in accordance with the principles of the Declaration of Helsinki and approved by the Institutional Review Board of the University of Texas Medical Branch (https://clinicaltrials.gov number NCT01661595, first posted on 8 August 2012). Written informed consent was obtained from all subjects prior to participation.

## Supporting information


Appendix S1.

## Data Availability

The data that support the findings of this study are available from the corresponding author upon reasonable request.
