# Supplementary material for: Assessing the therapeutic potential of PDE‐5 inhibitors in adults: Insights from a multi‐omics study
Source: Physiol Rep. 2026 Sep 21;14(18):e71104. doi: 10.14814/phy2.71104 (PMC13591550; doi:10.14814/phy2.71104)
Supplement: Supplementary file 1 [file PHY2-14-e71104-s001.docx]

**Supplementary Materials**

**Supplementary Methods**

**Physical performance**

*Handgrip maximum voluntary contraction and endurance*

All subjects underwent handgrip testing prior to and 80-90 minutes post pill ingestion at 0, 4, and 8 weeks. Determination of submaximal and maximal voluntary contraction (kg) was obtained with 3 sets of 2 contractions (1 dominant hand, 1 nondominant hand) at 25%, 50% and 100% perceived effort. Contractions were spaced 15 seconds apart, alternating arms (i.e. 30 second rest between contractions in each hand), with a 5 second hold for each contraction. The maximal force generated for each effort level in sets 2 and 3 was averaged. Following a five-minute rest, an endurance test was performed by holding a continuous contraction at 10% maximal force in the dominant arm for five minutes while the nondominant hand contracted for the last 5 seconds of every 30-second block, alternating between matching their dominant arm perceived effort and 25% perceived effort. Directly preceding and following the endurance portion of each handgrip test, subjects were asked to rate their fatigue from 0 (“no fatigue at all”) to 10 (“worst fatigue possible”) for their dominant forearm, nondominant forearm, and whole body. The peak force produced by the nondominant hand during 25% effort contractions during the endurance test was averaged and divided by the maximum force recorded during maximal voluntary contraction testing for 25% effort contractions of the nondominant hand as an index of fatigue.

*Leg extension torque, endurance and recovery*

Peak isometric and isokinetic torque (newton-meters - nM), endurance and recovery of the dominant leg knee extensors were assessed using a Biodex System 4 Pro dynamometer ~150 minutes post-pill ingestion during study visits at weeks 4 and 8 similar to previously detailed studies (Sheffield-Moore et al. 2013; Wright et al. 2020). Prior to testing, subjects performed 30 knee extensions with very low resistance as a warm-up. Next, subjects performed 3 sets of isometric contractions (0° per second, 5 second hold, joint angle 90°) at 25%, 50%, and 100% effort with 15 seconds rest between each contraction. Isometric torque was determined by averaging the peak torque generated during the final two sets for each effort level. After a 2-minute rest period, subjects performed 3 sets of isokinetic contractions (maximum angular velocity of 120° per second) at 25%, 50%, and 100% effort, with contractions spaced 5 seconds apart. Isokinetic torque was determined by averaging the peak torque produced during the final two sets for each level of effort. After a two-minute rest period, subjects performed an isokinetic endurance test. Subjects completed maximal effort isokinetic contractions at a maximal angular velocity of 120° per second for 50 repetitions. Endurance was assessed by summing the number of repetitions with torque ≥ 50% peak isokinetic torque achieved. To assess recovery, subjects performed 3 sets of isometric contractions (90° joint angle) at 0, 3, and 5 minutes after completing the isokinetic endurance test at 100%, 50%, and 25% maximal effort. Each contraction within a set was held for 5 seconds, with 1 second rest between contractions. Recovery torque production was calculated as percentage of isometric torque recorded prior to the endurance test for each effort level. All strength measurements were corrected for the influence of gravity using the Biodex software. Subjects were asked to rate their fatigue on a scale of 0 (“no fatigue at all”) to 10 (“worst possible”) for their dominant leg and whole body immediately preceding and immediately following Biodex testing.

*Walking performance*

Walking performance was assessed using a modified 6-minute walk test ~135 minutes post-pill ingestion during study visits at weeks 4 and 8. Subjects were asked to walk for 2 minutes each at perceived efforts of 25%, 50%, and 100% (Wright et al. 2020). Distance was measured for each effort level.

**Femoral artery blood flow**

Doppler ultrasound was used to measure leg hemodynamic responses ~2-3.5 hours pre-pill ingestion and 30 minutes post-pill ingestion during study visits at weeks 0, 4, and 8 using an ultrasound system (HDI-5000; Philips Medical Systems, Bothell, WA) and linear array transducer (L7-4) with a transmit frequency of 12MHz. Three electrocardiogram (ECG) leads were placed for heart rate and R wave monitoring. 2-D imaging of the common femoral artery was performed in the long axis. Images were triggered to the R wave of the cardiac cycle, and the femoral artery diameter was measured using online video calipers. A pulsed-wave Doppler sample blood volume was adjusted to insonate the entire artery, and mean blood velocity was measured using online angle correction and analysis software. Femoral artery mean blood flow was calculated from 2-D and Doppler ultrasound data using the equation: Q = vπ ∙ (d/2)^2^ ∙ 60, where Q is femoral blood flow (ml/min), v is mean femoral artery blood flow velocity (cm/s), and d is femoral artery diameter (cm).

**Body composition**

Body composition was assessed with Dual Energy X-Ray Absorptiometry (DEXA - GE Lunar iDXA) during study visits at weeks 0, 4, and 8.

**Subject-reported outcomes**

*Fatigue*

Subject-reported fatigue was assessed using the MD Anderson Brief Fatigue Inventory (BFI, a visual analog scale for fatigue) (Mendoza et al. 1999) and the Multidimensional Fatigue Symptom Inventory—Short Form (MFSI) (Stein et al. 2004). The BFI was completed daily by subjects over the course of the 8-week study period. The MFSI was completed during study visits at weeks 0, 4, and 8. The BFI is a 9-item questionnaire that assesses fatigue presently (“right now”), over the past 24 hours, and over the past week. The MFSI comprises 30 statements for which subjects rate the extent of their agreement. The items are rated on a 5-point Likert scale, with 0 indicating the statement is not at all true and 4 indicating the statement is very true with respect to their lives over the last 7 days. The individual item responses are used to calculate empirically-derived subscales assessing general, physical, emotional, and mental fatigue, in addition to vigor.

*Mood*

Mood was assessed using the 65-item Profile of Mood States—Short Form (POMS) questionnaire (Curran, Andrykowski, and Studts 1995), a validated measure that yields an overall score (range 0-168, with higher scores indicating more disturbance in mood) and 6 subscales (tension-anxiety, depression-dejection, anger-hostility, vigor-activity, fatigue-inertia, and confusion-bewilderment). The POMS was completed during study visits at weeks 0, 4, and 8.

**Supplementary Statistical Analyses**

*Physical performance*, *perceptual fatigue, and femoral artery blood flow*

Analyses were conducted using Graphpad Prism 10.6.1. Paired t-tests were used to compare measurements collected during pre-treatment (week 0 for S-P/T-P groups, and week 4 for P-S/P-T groups) and post-treatment study visits (week 4 for S-P/T-P groups, and week 8 for P-S/P-T groups). Biodex-related measures and walking performance were measured once each at the week 4 and week 8 study visit, but not during the week 0 study visit, so only the P-S/P-T groups were included in the analyses of these measures. For outcomes measured both pre- and post-pill, the pre-pill measurement was compared among timepoints.

Handgrip-related measures and femoral artery blood flow were additionally subjected to analyses of short-term effects of PDE-5 treatment since they were measured both pre- and post-pill at each study visit. Data were analyzed separately for treatment naïve (week 0 for S-P/T-P groups, no equivalent timepoint for P-S/P-T groups since they were given placebo at the week 4 study visit) and treated (week 4 for S-P/T-P groups, and week 8 for P-S/P-T groups) timepoints; paired t-tests were conducted to compare pre-pill and post-pill samples.

*Body composition and subject-reported outcomes*

Analyses were conducted using Graphpad Prism 10.6.1. Paired t-tests were used to compare pre-treatment (week 0 for S-P/T-P groups, and week 4 for P-S/P-T groups) and post-treatment values (week 4 for S-P/T-P groups, and week 8 for P-S/P-T groups) for body composition outcomes, MFSI, and POMS. Linear mixed effects models were used to analyze change in BFI scores over the 4-week duration of PDE-5 inhibitor treatment.

The Benjamin, Krieger, and Yekutieli method was used to control the false discovery rate (FDR, 0.1) for all pre- to post-treatment and pre- to post-pill comparisons conducted using Graphpad Prism 10.6.1. FDR-adjusted p values are reported as q values.

**Supplementary Figures, Tables, and Results**

Table S1. Metabolite (listed in numeroalphabetical order) log2(fold-changes) over four weeks of treatment with PDE-5 inhibitor. Metabolites marked with one asterisk were significantly different post-treatment compared to pre-treatment for either the sildenafil or tadalafil group. Metabolites marked with two asterisks were significantly different for both the sildenafil and tadalafil groups. Positive log2(fold-change) values indicate an increase in that metabolite following four weeks of treatment with PDE-5 inhibitor and negative log2(fold-change) values indicate a decrease in that metabolite following four weeks of treatment with PDE-5 inhibitor.

| Metabolite | Measured transition ions (parent / product) | Sildenafil | | Tadalafil | |
| --- | --- | --- | --- | --- | --- |
|  |  | log2(fold-change) | raw p-value | log2(fold-change) | raw p-value |
| 11,12-dihydroxyeicosatrienoic acid | 337.2 / 167.1 | 0.079 | 0.729 | 0.122 | 0.631 |
| 12-hydroxyeicosatetraenoic acid (12-HETE) ** | 319.2 / 179.1 | -0.693 | 0.034 | -0.743 | 0.018 |
| 13,14-dihydro-15-keto prostaglandin D2 | 351.2 / 207.1 | 0.057 | 0.834 | 0.037 | 0.891 |
| 13-hydroxyoctadecadienoic acid (13-HODE) * | 295.2 / 195.1 | -0.217 | 0.427 | -0.551 | 0.033 |
| 14,15-dihydroxyeicosatrienoic acid (14,15-DiHETrE) * | 337.2 / 207.1 | -0.340 | 0.240 | -0.565 | 0.034 |
| 15-keto prostaglandin F2α | 351.2 / 113.1 | filtered out with IQR estimate | | -0.155 | 0.556 |
| 20-hydroxy leukotriene B4 | 351.2 / 195.1 | -0.189 | 0.423 | filtered out with IQR estimate | |
| 2-arachidonoylglycerol | 379.4 / 287.2 | -0.160 | 0.560 | 0.051 | 0.854 |
| 5-hydroxyeicosatetraenoic acid (5-HETE) * | 319.2 / 115.1 | -0.544 | 0.048 | -0.348 | 0.190 |
| 9-hydroxyoctadecadienoic acid | 295.2 / 171.1 | -0.131 | 0.598 | -0.098 | 0.700 |
| Arachidonic acid | 310.1 / 76.9 | 0.119 | 0.646 | 0.227 | 0.365 |
| Adrenic acid | 331.2 / 287.1 | 0.247 | 0.414 | 0.097 | 0.706 |
| Argininosuccinic acid * | 289.1 / 131.1 | 0.501 | 0.146 | 0.711 | 0.015 |
| Citrulline * | 174.1 / 130.8 | 0.427 | 0.100 | 0.748 | 0.012 |
| Docosahexaenoic acid | 332.1 / 288.4 | -0.055 | 0.838 | -0.005 | 0.986 |
| Docosapentaenoic acid | 329.2 / 285.1 | -0.038 | 0.897 | 0.080 | 0.763 |
| Eicosapentaenoic acid | 306.1 / 262.3 | 0.005 | 0.987 | -0.157 | 0.562 |
| L-Arginine * | 175.1 / 70.0 | 0.635 | 0.022 | 0.563 | 0.059 |
| Linoleoylethanolamide | 324.4 / 62.1 | 0.165 | 0.584 | -0.074 | 0.796 |
| Leukotriene B4 | 335.2 / 195.1 | filtered out with IQR estimate | | filtered out with IQR estimate | |
| Maresin conjugates in tissue regeneration 2 | 521.3 / 191.1 | 0.023 | 0.925 | 0.137 | 0.577 |
| Maresin conjugates in tissue regeneration 3 | 464.3 / 191.1 | 0.240 | 0.488 | -0.310 | 0.384 |
| Oleoylethanolamide | 326.4 / 62.1 | 0.087 | 0.772 | 0.174 | 0.535 |
| Ornithine | 133.1 / 43.0 | 0.434 | 0.106 | 0.418 | 0.160 |
| Palmitoylethanolamide | 300.4 / 62.1 | 0.333 | 0.176 | 0.051 | 0.847 |
| Protectin D1 * | 359.2 / 153.1 | 0.055 | 0.836 | 1.030 | 0.001 |
| Protein conjugates in tissue regeneration 2 | 521.3 / 231.1 | 0.179 | 0.578 | 0.037 | 0.880 |
| Protein conjugates in tissue regeneration 3 | 464.3 / 231.1 | -0.017 | 0.954 | -0.441 | 0.233 |
| Prostaglandin J2 | 333.2 / 271.1 | 0.197 | 0.419 | 0.208 | 0.491 |
| Proline | 116.1 / 70.0 | 0.354 | 0.177 | 0.576 | 0.050 |
| Resolvin E1 | 349.2 / 195.1 | 0.182 | 0.435 | -0.076 | 0.754 |
| Stearoylethanolamide | 328.4 / 62.1 | 0.459 | 0.108 | 0.479 | 0.087 |

Table S2. Downregulated genes in skeletal muscle following four weeks of treatment with sildenafil.

| Gene symbol | Gene name | logFC | FDR |
| --- | --- | --- | --- |
| ADSS1 | adenylosuccinate synthase 1 | -0.4 | 0.052 |
| ANKRD23 | ankyrin repeat domain 23 | -0.5 | 0.097 |
| ASB10 | ankyrin repeat and SOCS box containing 10 | -0.48 | 0.04 |
| CTSF | cathepsin F | -0.39 | 0.088 |
| DNAAF9 | dynein axonemal assembly factor 9 | -0.61 | 0.068 |
| EEF1B2 | eukaryotic translation elongation factor 1 beta 2 | -0.34 | 0.097 |
| ENO3 | enolase 3 | -0.4 | 0.069 |
| FBP2 | fructose-bisphosphatase 2 | -0.38 | 0.079 |
| KLHL30 | kelch like family member 30 | -0.43 | 0.085 |
| *NA* | *NA* (unnamed, Ensembl ID ENSG00000267423) | -0.89 | 0.091 |
| *NA* | *NA* (unnamed, Ensembl ID ENSG00000260398) | -0.44 | 0.063 |
| NUDT3 | nudix hydrolase 3 | -0.29 | 0.087 |
| NXPE3 | neurexophilin and PC-esterase domain family member 3 | -0.43 | 0.052 |
| OPTN | optineurin | -0.35 | 0.064 |
| PDP1 | pyruvate dehydrogenase phosphatase catalytic subunit 1 | -0.39 | 0.068 |
| PDZRN3 | PDZ domain containing ring finger 3 | -0.42 | 0.097 |
| PEBP4 | phosphatidylethanolamine binding protein 4 | -0.42 | 0.097 |
| SLC1A4 | solute carrier family 1 member 4 | -0.57 | 0.073 |
| SLC25A30 | solute carrier family 25 member 30 | -0.67 | 0.084 |
| SYNPO | synaptopodin | -0.35 | 0.1 |
| TBC1D25 | TBC1 domain family member 25 | -0.8 | 0.087 |
| TMEM70 | transmembrane protein 70 | -0.52 | 0.069 |
| UPK3A | uroplakin 3A | -0.86 | 0.018 |

Table S3. Upregulated genes in skeletal muscle following four weeks of treatment with sildenafil.

| Gene symbol | Gene name | logFC | FDR |
| --- | --- | --- | --- |
| ADD3 | adducin 3 | 0.7 | 0.04 |
| AHNAK2 | AHNAK nucleoprotein 2 | 1.26 | 0.076 |
| ANXA1 | annexin A1 | 0.83 | 0.091 |
| ARHGEF2 | Rho/Rac guanine nucleotide exchange factor 2 | 0.91 | 0.063 |
| ARPC1B | actin related protein 2/3 complex subunit 1B | 0.96 | 0.063 |
| ASPN | asporin | 2.45 | 0.068 |
| BASP1 | brain abundant membrane attached signal protein 1 | 1.36 | 0.052 |
| C1QA | complement C1q A chain | 0.76 | 0.068 |
| C1QC | complement C1q C chain | 0.89 | 0.085 |
| CCDC80 | coiled-coil domain containing 80 | 1.17 | 0.088 |
| CCN5 | cellular communication network factor 5 | 0.63 | 0.068 |
| CD302 | CD302 molecule | 1.34 | 0.068 |
| CD68 | CD68 molecule | 1.2 | 0.04 |
| CD82 | CD82 molecule | 1.03 | 0.04 |
| CDC42SE1 | CDC42 small effector 1 | 1 | 0.068 |
| CFH | complement factor H | 0.94 | 0.047 |
| CILP | cartilage intermediate layer protein | 0.88 | 0.097 |
| CMTM3 | CKLF like MARVEL transmembrane domain containing 3 | 1.3 | 0.04 |
| COL14A1 | collagen type XIV alpha 1 chain | 1.37 | 0.097 |
| COL5A1 | collagen type V alpha 1 chain | 1.41 | 0.069 |
| COL5A2 | collagen type V alpha 2 chain | 1.82 | 0.088 |
| COTL1 | coactosin like F-actin binding protein 1 | 1.02 | 0.087 |
| CSF1R | colony stimulating factor 1 receptor | 0.73 | 0.087 |
| CSF3R | colony stimulating factor 3 receptor | 1.43 | 0.089 |
| CTSK | cathepsin K | 1.12 | 0.098 |
| CYBB | cytochrome b-245 beta chain | 0.91 | 0.052 |
| DCLK1 | doublecortin like kinase 1 | 2.02 | 0.069 |
| DCN | decorin | 0.55 | 0.069 |
| DDR2 | discoidin domain receptor tyrosine kinase 2 | 0.79 | 0.087 |
| EEF1A1 | eukaryotic translation elongation factor 1 alpha 1 | 0.63 | 0.064 |
| EFEMP1 | EGF containing fibulin extracellular matrix protein 1 | 0.95 | 0.047 |
| EMP1 | epithelial membrane protein 1 | 0.79 | 0.098 |
| ERBB3 | erb-b2 receptor tyrosine kinase 3 | 1.79 | 0.068 |
| F13A1 | coagulation factor XIII A chain | 1.38 | 0.04 |
| FAM110A | family with sequence similarity 110 member A | 1.27 | 0.052 |
| FBLN2 | fibulin 2 | 0.61 | 0.068 |
| FBN1 | fibrillin 1 | 0.95 | 0.087 |
| FGGY | FGGY carbohydrate kinase domain containing | 0.63 | 0.047 |
| FNDC1 | fibronectin type III domain containing 1 | 1.55 | 0.04 |
| FOS | Fos proto-oncogene, AP-1 transcription factor subunit | 1.85 | 0.035 |
| FSTL1 | follistatin like 1 | 1 | 0.014 |
| GBP2 | guanylate binding protein 2 | 0.63 | 0.087 |
| GJA1 | gap junction protein alpha 1 | 1.5 | 0.035 |
| GPNMB | glycoprotein nmb | 0.68 | 0.085 |
| GTF2F1 | general transcription factor IIF subunit 1 | 0.55 | 0.087 |
| HTRA1 | HtrA serine peptidase 1 | 1 | 0.052 |
| ITGA5 | integrin subunit alpha 5 | 1.14 | 0.087 |
| ITM2A | integral membrane protein 2A | 0.54 | 0.097 |
| JDP2 | Jun dimerization protein 2 | 0.83 | 0.091 |
| LAMA4 | laminin subunit alpha 4 | 0.82 | 0.069 |
| LAMP1 | lysosomal associated membrane protein 1 | 0.52 | 0.068 |
| LAPTM5 | lysosomal protein transmembrane 5 | 1.35 | 0.035 |
| LILRB5 | leukocyte immunoglobulin like receptor B5 | 1.01 | 0.084 |
| LRP1 | LDL receptor related protein 1 | 0.57 | 0.068 |
| LRRC17 | leucine rich repeat containing 17 | 1.44 | 0.052 |
| MANF | mesencephalic astrocyte derived neurotrophic factor | 0.96 | 0.097 |
| MMP14 | matrix metallopeptidase 14 | 1.23 | 0.058 |
| MMP2 | matrix metallopeptidase 2 | 1.13 | 0.047 |
| MNDA | myeloid cell nuclear differentiation antigen | 1.66 | 0.068 |
| MPZL1 | myelin protein zero like 1 | 0.89 | 0.084 |
| MRC2 | mannose receptor C-type 2 | 0.72 | 0.033 |
| MXRA5 | matrix remodeling associated 5 | 2.45 | 0.042 |
| MXRA8 | matrix remodeling associated 8 | 0.76 | 0.064 |
| NBL1 | NBL1, DAN family BMP antagonist | 0.97 | 0.055 |
| NCF2 | neutrophil cytosolic factor 2 | 2.13 | 0.04 |
| OGN | osteoglycin | 1.69 | 0.068 |
| PADI2 | peptidyl arginine deiminase 2 | 0.49 | 0.052 |
| PAG1 | phosphoprotein membrane anchor with glycosphingolipid microdomains 1 | 1.02 | 0.086 |
| PLAC9 | placenta associated 9 | 0.74 | 0.052 |
| PLEK | pleckstrin | 1.51 | 0.068 |
| POSTN | periostin | 4.2 | 0.068 |
| PPBP | pro-platelet basic protein | 2.05 | 0.069 |
| PPIB | peptidylprolyl isomerase B | 0.69 | 0.087 |
| PPP1R18 | protein phosphatase 1 regulatory subunit 18 | 1.19 | 0.068 |
| PRG4 | proteoglycan 4 | 1.31 | 0.079 |
| PRKCB | protein kinase C beta | 1.11 | 0.087 |
| RHOC | ras homolog family member C | 0.72 | 0.068 |
| RNY4 | RNA, Ro60-associated Y4 | 0.99 | 0.04 |
| S100A10 | S100 calcium binding protein A10 | 0.91 | 0.097 |
| S100A8 | S100 calcium binding protein A8 | 1.61 | 0.033 |
| SAA1 | serum amyloid A1 | 4.86 | 0.064 |
| SAA2 | serum amyloid A2 | 6.33 | 0.014 |
| SERPINH1 | serpin family H member 1 | 1.14 | 0.08 |
| SESN3 | sestrin 3 | 1.15 | 0.068 |
| SFRP2 | secreted frizzled related protein 2 | 2.31 | 0.076 |
| SH3D19 | SH3 domain containing 19 | 0.59 | 0.097 |
| SH3PXD2B | SH3 and PX domains 2B | 1.71 | 0.064 |
| SLPI | secretory leukocyte peptidase inhibitor | 1.64 | 0.087 |
| SOX4 | SRY-box transcription factor 4 | 1.16 | 0.068 |
| SPTLC2 | serine palmitoyltransferase long chain base subunit 2 | 0.93 | 0.068 |
| STMN1 | stathmin 1 | 1.06 | 0.068 |
| TGFBI | transforming growth factor beta induced | 0.94 | 0.068 |
| TGFBR2 | transforming growth factor beta receptor 2 | 0.72 | 0.04 |
| TGFBR3 | transforming growth factor beta receptor 3 | 0.66 | 0.097 |
| TIMP2 | TIMP metallopeptidase inhibitor 2 | 0.7 | 0.068 |
| TMSB10 | thymosin beta 10 | 0.69 | 0.069 |
| TUBB | tubulin beta class I | 0.52 | 0.087 |
| TYMP | thymidine phosphorylase | 1.15 | 0.049 |
| VTRNA1-3 | vault RNA 1-3 | 3.41 | 0.033 |
| ZFP36L2 | ZFP36 ring finger protein like 2 | 0.46 | 0.068 |
| ZNF217 | zinc finger protein 217 | 0.87 | 0.087 |

Table S4. Mixed effects models results for chronic effects of PDE-5 inhibitor treatment with week 0 measurement included as covariate (following Method II from Mehrotra 2014). P-S: Placebo-Sildenafil, S-P: Sildenafil-Placebo, P-T: Placebo-Tadalafil, T-P: Tadalafil-Placebo. Two subjects from the P-S group and one subject from the S-P group did not have Week 0 measurements due to insufficient or damaged tissue, and as a result were excluded from the model. The “Missing Values” column indicates percent of missing response values (Week 4 and Week 8 measurements).

|  | Week 0 Measurement  p-value | Sequence  p-value | Period  p-value | Treatment  p-value | Week 0 Measurement * Period  p-value | # Missing Values/ # Total Response Values (%) | N  (P-S/ S-P/ P-T/ T-P) |
| --- | --- | --- | --- | --- | --- | --- | --- |
| Total Macrophages | 0.3437 | 0.7351 | 0.9606 | 0.3281 | 0.9855 | 7/26 (26.9%) | 2/3/4/4 |
| M1 Macrophages | 0.2168 | 0.253 | 0.2503 | 0.0627 | 0.3539 |  |  |
| M2 Macrophages | 0.6656 | 0.8948 | 0.4638 | 0.2806 | 0.4595 |  |  |
| M2:M1 Ratio | 0.1168 | 0.8859 | 0.9618 | 0.6663 | 0.5556 |  |  |

Table S5. Downregulated proteins in skeletal muscle following four weeks of treatment with tadalafil.

| Gene symbol | Gene name | UniProt ID | logFC | raw p |
| --- | --- | --- | --- | --- |
| *ACTN3* | actinin alpha 3 | Q08043 | -0.286 | 0.034 |
| *ACTR3* | actin related protein 3 | P61158 | -3.45 | 0.006 |
| *ADPRHL1* | ADP-ribosylhydrolase like 1 | Q8NDY3 | -1.27 | 0.021 |
| *ADSS1* | adenylosuccinate synthase 1 | Q8N142 | -0.81 | 0.034 |
| *AGL* | amylo-alpha-1,6-glucosidase and 4-alpha-glucanotransferase | P35573 | -0.386 | 0.018 |
| *AIFM1* | apoptosis inducing factor mitochondria associated 1 | O95831 | -0.253 | 0.047 |
| *ALDH1L1* | aldehyde dehydrogenase 1 family member L1 | O75891 | -0.904 | 0.023 |
| *ALDH1L2* | aldehyde dehydrogenase 1 family member L2 | Q3SY69 | -0.441 | 0.027 |
| *ALDOC* | aldolase, fructose-bisphosphate C | P09972 | -0.862 | 0.04 |
| *ANXA1* | annexin A1 | P04083 | -1.78 | 0.03 |
| *ANXA2* | annexin A2 | P07355 | -0.873 | 0.026 |
| *ANXA4* | annexin A4 | P09525 | -1.07 | 0.011 |
| *AP2M1* | adaptor related protein complex 2 subunit mu 1 | Q96CW1 | -1.07 | 0.014 |
| *ARHGDIB* | Rho GDP dissociation inhibitor beta | P52566 | -2.41 | 0.006 |
| *ATP5F1B* | ATP synthase F1 subunit beta | P06576 | -0.168 | 0.037 |
| *ATP5MG* | ATP synthase membrane subunit g | O75964 | -0.143 | 0.01 |
| *ATP6V1H* | ATPase H+ transporting V1 subunit H | Q9UI12 | -1.79 | 0.023 |
| *CAB39* | calcium binding protein 39 | Q9Y376 | -1.01 | 0.024 |
| *CAMK2B* | calcium/calmodulin dependent protein kinase II beta | Q13554 | -0.551 | 0.015 |
| *CAMK2G* | calcium/calmodulin dependent protein kinase II gamma | Q13555 | -0.342 | 0.048 |
| *CAPZA1* | capping actin protein of muscle Z-line subunit alpha 1 | P52907 | -0.624 | 0.015 |
| *CAPZA2* | capping actin protein of muscle Z-line subunit alpha 2 | P47755 | -0.455 | 0.006 |
| *CHCHD3* | coiled-coil-helix-coiled-coil-helix domain containing 3 | Q9NX63 | -0.621 | 0.023 |
| *CLTCL1* | clathrin heavy chain like 1 | P53675 | -1.21 | 0.022 |
| *COBL* | cordon-bleu WH2 repeat protein | O75128 | -1.61 | 0.048 |
| *COL6A1* | collagen type VI alpha 1 chain | P12109 | -0.932 | 0.024 |
| *COQ3* | coenzyme Q3, methyltransferase | Q9NZJ6 | -1.14 | 0.033 |
| *COQ5* | coenzyme Q5, methyltransferase | Q5HYK3 | -1.29 | 0.018 |
| *CPNE2* | copine 2 | Q96FN4 | -0.631 | 0.034 |
| *CRAT* | carnitine O-acetyltransferase | P43155 | -0.586 | 0.029 |
| *CRIP2* | cysteine rich protein 2 | P52943 | -0.658 | 0.043 |
| *CTNNB1* | catenin beta 1 | P35222 | -2.14 | 0.005 |
| *CUL4A* | cullin 4A | Q13619 | -0.715 | 0.043 |
| *DLAT* | dihydrolipoamide S-acetyltransferase | P10515 | -0.536 | 0.026 |
| *DYNC1I2* | dynein cytoplasmic 1 intermediate chain 2 | Q13409 | -0.767 | 0.045 |
| *EIF3A* | eukaryotic translation initiation factor 3 subunit A | Q14152 | -0.944 | 0.043 |
| *EIF3D* | eukaryotic translation initiation factor 3 subunit D | O15371 | -0.735 | 0.015 |
| *EIF4G1* | eukaryotic translation initiation factor 4 gamma 1 | Q04637 | -0.955 | 0.041 |
| *ESD* | esterase D | P10768 | -0.519 | 0.017 |
| *ESYT1* | extended synaptotagmin 1 | Q9BSJ8 | -1.59 | 0.05 |
| *GAA* | alpha glucosidase | P10253 | -1.52 | 0.044 |
| *GALM* | galactose mutarotase | Q96C23 | -1.6 | 0.05 |
| *GGCT* | gamma-glutamylcyclotransferase | O75223 | -1.54 | 0.023 |
| *GPD2* | glycerol-3-phosphate dehydrogenase 2 | P43304 | -0.491 | 0.016 |
| *GPX1* | glutathione peroxidase 1 | P07203 | -1.65 | 0.014 |
| *GSTO1* | glutathione S-transferase omega 1 | P78417 | -0.27 | 0.049 |
| *HNRNPU* | heterogeneous nuclear ribonucleoprotein U | Q00839 | -0.841 | 0.002 |
| *HSD17B10* | hydroxysteroid 17-beta dehydrogenase 10 | Q99714 | -0.552 | 0.044 |
| *HSD17B12* | hydroxysteroid 17-beta dehydrogenase 12 | Q53GQ0 | -1.03 | 0.024 |
| *HSP90B1* | heat shock protein 90 beta family member 1 | P14625 | -0.52 | 0.007 |
| *HSPB2* | heat shock protein family B (small) member 2 | Q16082 | -1.57 | 0.022 |
| *HSPB3* | heat shock protein family B (small) member 3 | Q12988 | -1.42 | 0.04 |
| *IDH2* | isocitrate dehydrogenase (NADP(+)) 2 | P48735 | -0.239 | 0.012 |
| *ILF2* | interleukin enhancer binding factor 2 | Q12905 | -0.733 | 0.017 |
| *IPO5* | importin 5 | O00410 | -0.975 | 0.047 |
| *KPNA1* | karyopherin subunit alpha 1 | P52294 | -0.87 | 0.033 |
| *KPNA6* | karyopherin subunit alpha 6 | O60684 | -0.398 | 0.036 |
| *L2HGDH* | L-2-hydroxyglutarate dehydrogenase | Q9H9P8 | -0.737 | 0.038 |
| *LAMA4* | laminin subunit alpha 4 | Q16363 | -1.22 | 0.043 |
| *LAMA5* | laminin subunit alpha 5 | O15230 | -1.48 | 0.032 |
| *LDHA* | lactate dehydrogenase A | P00338 | -0.277 | 0.005 |
| *LNPK* | lunapark, ER junction formation factor | Q9C0E8 | -2.87 | 0.027 |
| *LUM* | lumican | P51884 | -1.29 | 0.031 |
| *MACROH2A1* | macroH2A.1 histone | O75367 | -0.667 | 0.035 |
| *MRPL16* | mitochondrial ribosomal protein L16 | Q9NX20 | -0.84 | 0.036 |
| *NAMPT* | nicotinamide phosphoribosyltransferase | P43490 | -1 | 0.004 |
| *NDRG2* | NDRG family member 2 | Q9UN36 | -0.231 | 0.042 |
| *NDUFA2* | NADH:ubiquinone oxidoreductase subunit A2 | O43678 | -0.346 | 0.03 |
| *NDUFS8* | NADH:ubiquinone oxidoreductase core subunit S8 | O00217 | -0.85 | 0.012 |
| *P4HB* | prolyl 4-hydroxylase subunit beta | P07237 | -0.147 | 0.039 |
| *PAFAH1B1* | platelet activating factor acetylhydrolase 1b regulatory subunit 1 | P43034 | -0.458 | 0.041 |
| *PDIA3* | protein disulfide isomerase family A member 3 | P30101 | -1.04 | 0.035 |
| *PEPD* | peptidase D | P12955 | -1.8 | 0.034 |
| *PFN1* | profilin 1 | P07737 | -0.44 | 0.023 |
| *PGM1* | phosphoglucomutase 1 | P36871 | -0.37 | 0.039 |
| *PHKB* | phosphorylase kinase regulatory subunit beta | Q93100 | -0.275 | 0.014 |
| *PIN1* | peptidylprolyl cis/trans isomerase, NIMA-interacting 1 | Q13526 | -0.612 | 0.009 |
| *PKLR* | pyruvate kinase L/R | P30613 | -0.961 | 0.043 |
| *PKM* | pyruvate kinase M1/2 | P14618 | -0.25 | 0.033 |
| *PPIA* | peptidylprolyl isomerase A | P62937 | -0.759 | 0.044 |
| *PPP2CA* | protein phosphatase 2 catalytic subunit alpha | P67775 | -0.76 | 0.017 |
| *PRKAB2* | protein kinase AMP-activated non-catalytic subunit beta 2 | O43741 | -0.999 | 0.024 |
| *PRKAR1A* | protein kinase cAMP-dependent type I regulatory subunit alpha | P10644 | -0.284 | 0.027 |
| *PRKDC* | protein kinase, DNA-activated, catalytic subunit | P78527 | -1.3 | 0.048 |
| *PSMB1* | proteasome 20S subunit beta 1 | P20618 | -0.888 | 0.016 |
| *PSMB3* | proteasome 20S subunit beta 3 | P49720 | -0.823 | 0.025 |
| *PSMC3* | proteasome 26S subunit, ATPase 3 | P17980 | -0.33 | 0.048 |
| *PSMD7* | proteasome 26S subunit, non-ATPase 7 | P51665 | -1.21 | 0.027 |
| *PYGM* | glycogen phosphorylase, muscle associated | P11217 | -0.302 | 0.025 |
| *RNF123* | ring finger protein 123 | Q5XPI4 | -0.372 | 0.048 |
| *RPL18* | ribosomal protein L18 | Q07020 | -0.125 | 0.017 |
| *RPL7A* | ribosomal protein L7a | P62424 | -0.776 | 0.042 |
| *RPL9* | ribosomal protein L9 | P32969 | -1.76 | 0.026 |
| *RPN1* | ribophorin I | P04843 | -0.92 | 0.027 |
| *RPN2* | ribophorin II | P04844 | -1.85 | 0.017 |
| *RPS23* | ribosomal protein S23 | P62266 | -0.908 | 0.012 |
| *RPS24* | ribosomal protein S24 | P62847 | -0.429 | 0.038 |
| *RYR1* | ryanodine receptor 1 | P21817 | -0.55 | 0.03 |
| *RYR3* | ryanodine receptor 3 | Q15413 | -0.308 | 0.048 |
| *SAMM50* | SAMM50 sorting and assembly machinery component | Q9Y512 | -0.582 | 0.008 |
| *SBDS* | SBDS ribosome maturation factor | Q9Y3A5 | -0.789 | 0.037 |
| *SEPTIN2* | septin 2 | Q15019 | -1.38 | 0.035 |
| *SEPTIN6* | septin 6 | Q14141 | -0.657 | 0.039 |
| *SERPINB6* | serpin family B member 6 | P35237 | -1.13 | 0.004 |
| *SGCG* | sarcoglycan gamma | Q13326 | -0.66 | 0.024 |
| *SKIC3* | SKI3 subunit of superkiller complex | Q6PGP7 | -1.04 | 0.039 |
| *SLC25A3* | solute carrier family 25 member 3 | Q00325 | -0.281 | 0.013 |
| *TMEM43* | transmembrane protein 43 | Q9BTV4 | -0.543 | 0.049 |
| *TMT1A* | thiol methyltransferase 1A | Q9H8H3 | -0.749 | 0.009 |
| *TRIM72* | tripartite motif containing 72 | Q6ZMU5 | -0.861 | 0.044 |
| *TUBA8* | tubulin alpha 8 | Q9NY65 | -0.345 | 0.029 |
| *TUBB1* | tubulin beta 1 class VI | Q9H4B7 | -0.742 | 0.034 |
| *TUBB3* | tubulin beta 3 class III | Q13509 | -0.323 | 0.027 |
| *TUBB6* | tubulin beta 6 class V | Q9BUF5 | -0.281 | 0.02 |
| *USP14* | ubiquitin specific peptidase 14 | P54578 | -0.556 | 0.008 |
| *XPNPEP1* | X-prolyl aminopeptidase 1 | Q9NQW7 | -1.28 | 0.017 |

Table S6. Upregulated proteins in skeletal muscle following four weeks of treatment with tadalafil.

| Gene symbol | Gene name | UniProt ID | logFC | raw p |
| --- | --- | --- | --- | --- |
| *MECR* | mitochondrial trans-2-enoyl-CoA reductase | Q9BV79 | 1.64 | 0.022 |
| *PSMC4* | proteasome 26S subunit, ATPase 4 | P43686 | 3.79 | 0.042 |

Table S7. Downregulated proteins in skeletal muscle following four weeks of treatment with sildenafil.

| Gene symbol | Gene name | UniProt ID | logFC | raw p |
| --- | --- | --- | --- | --- |
| COQ3 | coenzyme Q3, methyltransferase | Q9NZJ6 | -1.45 | 0.013 |
| DBT | dihydrolipoamide branched chain transacylase E2 | P11182 | -0.864 | 0.033 |
| DHRS7C | dehydrogenase/reductase 7C | A6NNS2 | -1.38 | 0.018 |
| HINT3 | histidine triad nucleotide binding protein 3 | Q9NQE9 | -0.315 | 0.022 |
| MFN2 | mitofusin 2 | O95140 | -2.21 | 0.047 |
| NIPSNAP2 | nipsnap homolog 2 | O75323 | -0.468 | 0.02 |
| NLRX1 | NLR family member X1 | Q86UT6 | -0.894 | 0.024 |
| PHKA1 | phosphorylase kinase regulatory subunit alpha 1 | P46020 | -0.657 | 0.009 |
| UBE2D3 | ubiquitin conjugating enzyme E2 D3 | P61077 | -0.839 | 0.045 |
| VDAC3 | voltage dependent anion channel 3 | Q9Y277 | -0.356 | 0.044 |

Table S8. Upregulated proteins in skeletal muscle following four weeks of treatment with sildenafil.

| Gene symbol | Gene name | UniProt ID | logFC | raw p |
| --- | --- | --- | --- | --- |
| AARS1 | alanyl-tRNA synthetase 1 | P49588 | 1.29 | 0.024 |
| ABLIM2 | actin binding LIM protein family member 2 | Q6H8Q1 | 0.705 | 0.027 |
| ACAA2 | acetyl-CoA acyltransferase 2 | P42765 | 0.36 | 0.003 |
| ACSL3 | acyl-CoA synthetase long chain family member 3 | O95573 | 0.983 | 0.045 |
| AHSA1 | activator of HSP90 ATPase activity 1 | O95433 | 0.958 | 0.034 |
| AK2 | adenylate kinase 2 | P54819 | 0.794 | 0.046 |
| ALB | albumin | P02768 | 0.696 | 0.04 |
| ALDH1L2 | aldehyde dehydrogenase 1 family member L2 | Q3SY69 | 1.28 | 0.0063 |
| ALDH9A1 | aldehyde dehydrogenase 9 family member A1 | P49189 | 0.41 | 0.0083 |
| AP2A1 | adaptor related protein complex 2 subunit alpha 1 | O95782 | 1.8 | 0.043 |
| BLVRA | biliverdin reductase A | P53004 | 1.07 | 0.033 |
| BPGM | bisphosphoglycerate mutase | P07738 | 1.42 | 0.013 |
| CA2 | carbonic anhydrase 2 | P00918 | 0.337 | 0.032 |
| CACYBP | calcyclin binding protein | Q9HB71 | 1.04 | 0.039 |
| CAMK2A | calcium/calmodulin dependent protein kinase II alpha | Q9UQM7 | 0.459 | 0.042 |
| CAMK2B | calcium/calmodulin dependent protein kinase II beta | Q13554 | 0.347 | 0.024 |
| CAPZB | capping actin protein of muscle Z-line subunit beta | P47756 | 0.277 | 0.00042 |
| CASQ2 | calsequestrin 2 | O14958 | 0.294 | 0.03 |
| CAV1 | caveolin 1 | Q03135 | 0.794 | 0.016 |
| CAVIN1 | caveolae associated protein 1 | Q6NZI2 | 0.797 | 0.045 |
| CCT2 | chaperonin containing TCP1 subunit 2 | P78371 | 0.499 | 0.005 |
| CCT4 | chaperonin containing TCP1 subunit 4 | P50991 | 0.969 | 0.0058 |
| CCT5 | chaperonin containing TCP1 subunit 5 | P48643 | 0.982 | 0.021 |
| CCT8 | chaperonin containing TCP1 subunit 8 | P50990 | 0.482 | 0.032 |
| CD36 | CD36 molecule (CD36 blood group) | P16671 | 0.668 | 0.017 |
| CDH13 | cadherin 13 | P55290 | 0.494 | 0.013 |
| COBL | cordon-bleu WH2 repeat protein | O75128 | 0.264 | 0.044 |
| COPS2 | COP9 signalosome subunit 2 | P61201 | 0.653 | 0.02 |
| COPS4 | COP9 signalosome subunit 4 | Q9BT78 | 0.915 | 0.0081 |
| CORO6 | coronin 6 | Q6QEF8 | 0.77 | 0.05 |
| CRMP1 | collapsin response mediator protein 1 | Q14194 | 0.57 | 0.037 |
| CRYAB | crystallin alpha B | P02511 | 0.32 | 0.03 |
| CUL2 | cullin 2 | Q13617 | 0.636 | 0.027 |
| CUL4A | cullin 4A | Q13619 | 0.823 | 0.045 |
| CYB5R3 | cytochrome b5 reductase 3 | P00387 | 0.161 | 0.032 |
| CYRIB | CYFIP related Rac1 interactor B | Q9NUQ9 | 0.919 | 0.029 |
| DES | desmin | P17661 | 0.175 | 0.033 |
| DNAJA2 | DnaJ heat shock protein family (Hsp40) member A2 | O60884 | 0.995 | 0.0036 |
| DNPEP | aspartyl aminopeptidase | Q9ULA0 | 0.925 | 0.0047 |
| DYSF | dysferlin | O75923 | 0.413 | 0.048 |
| ECPAS | Ecm29 proteasome adaptor and scaffold | Q5VYK3 | 0.811 | 0.017 |
| EIF1AX | eukaryotic translation initiation factor 1A X-linked | P47813 | 0.825 | 0.013 |
| EIF2S3 | eukaryotic translation initiation factor 2 subunit gamma | P41091 | 0.27 | 0.014 |
| EIF3B | eukaryotic translation initiation factor 3 subunit B | P55884 | 0.834 | 0.047 |
| EIF3C | eukaryotic translation initiation factor 3 subunit C | Q99613 | 1.07 | 0.0031 |
| EIF4A2 | eukaryotic translation initiation factor 4A2 | Q14240 | 0.644 | 0.028 |
| F13A1 | coagulation factor XIII A chain | P00488 | 2.29 | 0.041 |
| FAH | fumarylacetoacetate hydrolase | P16930 | 0.233 | 0.015 |
| FERMT2 | FERM domain containing kindlin 2 | Q96AC1 | 1.29 | 0.0097 |
| FKBP1A | FKBP prolyl isomerase 1A | P62942 | 0.857 | 0.048 |
| FLNC | filamin C | Q14315 | 0.26 | 0.016 |
| FLOT1 | flotillin 1 | O75955 | 1.79 | 0.032 |
| FXR2 | FMR1 autosomal homolog 2 | P51116 | 0.359 | 0.03 |
| GBE1 | 1,4-alpha-glucan branching enzyme 1 | Q04446 | 0.781 | 0.045 |
| GCLM | glutamate-cysteine ligase modifier subunit | P48507 | 1.52 | 0.046 |
| GDI2 | GDP dissociation inhibitor 2 | P50395 | 0.687 | 0.044 |
| GET3 | guided entry of tail-anchored proteins factor 3, ATPase | O43681 | 1.27 | 0.01 |
| GNB1 | G protein subunit beta 1 | P62873 | 0.544 | 0.025 |
| GNB2 | G protein subunit beta 2 | P62879 | 0.619 | 0.02 |
| GPS1 | G protein pathway suppressor 1 | Q13098 | 0.555 | 0.025 |
| GPX1 | glutathione peroxidase 1 | P07203 | 1.11 | 0.046 |
| GRB2 | growth factor receptor bound protein 2 | P62993 | 0.598 | 0.03 |
| H1-10 | H1.10 linker histone | Q92522 | 0.547 | 0.044 |
| HADHB | hydroxyacyl-CoA dehydrogenase trifunctional multienzyme complex subunit beta | P55084 | 0.206 | 0.026 |
| HNRNPA2B1 | heterogeneous nuclear ribonucleoprotein A2/B1 | P22626 | 0.85 | 0.019 |
| HNRNPR | heterogeneous nuclear ribonucleoprotein R | O43390 | 0.42 | 0.029 |
| HNRNPU | heterogeneous nuclear ribonucleoprotein U | Q00839 | 0.592 | 0.041 |
| HOMER1 | homer scaffold protein 1 | Q86YM7 | 1.21 | 0.038 |
| HSP90AA1 | heat shock protein 90 alpha family class A member 1 | P07900 | 0.644 | 0.01 |
| HSP90AB1 | heat shock protein 90 alpha family class B member 1 | P08238 | 0.658 | 0.012 |
| HSP90B1 | heat shock protein 90 beta family member 1 | P14625 | 0.663 | 0.04 |
| HSPA1A | heat shock protein family A (Hsp70) member 1A | P0DMV9 | 0.331 | 0.0027 |
| HSPA2 | heat shock protein family A (Hsp70) member 2 | P54652 | 0.423 | 0.0088 |
| HSPA4 | heat shock protein family A (Hsp70) member 4 | P34932 | 0.606 | 0.002 |
| HSPA8 | heat shock protein family A (Hsp70) member 8 | P11142 | 0.364 | 0.012 |
| HSPB3 | heat shock protein family B (small) member 3 | Q12988 | 1.37 | 0.03 |
| IGHM | immunoglobulin heavy constant mu | P01871 | 1.12 | 0.025 |
| IPO5 | importin 5 | O00410 | 0.956 | 0.042 |
| LAMA2 | laminin subunit alpha 2 | P24043 | 0.471 | 0.022 |
| LIMCH1 | LIM and calponin homology domains 1 | Q9UPQ0 | 2.81 | 0.0079 |
| LMAN1 | lectin, mannose binding 1 | P49257 | 0.497 | 0.04 |
| LMOD2 | leiomodin 2 | Q6P5Q4 | 1.59 | 0.00077 |
| MAP4 | microtubule associated protein 4 | P27816 | 0.465 | 0.046 |
| MAP7D1 | MAP7 domain containing 1 | Q3KQU3 | 0.566 | 0.017 |
| MLIP | muscular LMNA interacting protein | Q5VWP3 | 1.17 | 0.0042 |
| MTCH2 | mitochondrial carrier 2 | Q9Y6C9 | 0.366 | 0.046 |
| MYO1C | myosin IC | O00159 | 0.942 | 0.042 |
| NCLN | nicalin | Q969V3 | 1.05 | 0.035 |
| NID2 | nidogen 2 | Q14112 | 1.21 | 0.035 |
| NXN | nucleoredoxin | Q6DKJ4 | 1.82 | 0.012 |
| P4HB | prolyl 4-hydroxylase subunit beta | P07237 | 0.28 | 0.038 |
| PADI2 | peptidyl arginine deiminase 2 | Q9Y2J8 | 0.703 | 0.014 |
| PAFAH1B1 | platelet activating factor acetylhydrolase 1b regulatory subunit 1 | P43034 | 0.679 | 0.042 |
| PCBP2 | poly(rC) binding protein 2 | Q15366 | 1.13 | 0.024 |
| PDCD6IP | programmed cell death 6 interacting protein | Q8WUM4 | 1.37 | 0.012 |
| PFN2 | profilin 2 | P35080 | 0.594 | 0.026 |
| PRKACA | protein kinase cAMP-activated catalytic subunit alpha | P17612 | 0.176 | 0.041 |
| PRKAG1 | protein kinase AMP-activated non-catalytic subunit gamma 1 | P54619 | 1.74 | 0.03 |
| PRMT1 | protein arginine methyltransferase 1 | Q99873 | 0.537 | 0.031 |
| PSMA1 | proteasome 20S subunit alpha 1 | P25786 | 0.378 | 0.039 |
| PSMA6 | proteasome 20S subunit alpha 6 | P60900 | 0.306 | 0.032 |
| PSMD2 | proteasome 26S subunit ubiquitin receptor, non-ATPase 2 | Q13200 | 0.637 | 0.0033 |
| RAC1 | Rac family small GTPase 1 | P63000 | 0.426 | 0.019 |
| RAN | RAN, member RAS oncogene family | P62826 | 0.483 | 0.0055 |
| RAP1A | RAP1A, member of RAS oncogene family | P62834 | 0.569 | 0.044 |
| RNH1 | ribonuclease/angiogenin inhibitor 1 | P13489 | 0.929 | 0.024 |
| RPL18 | ribosomal protein L18 | Q07020 | 0.507 | 0.025 |
| RPL23 | ribosomal protein L23 | P62829 | 0.892 | 0.017 |
| RPL23A | ribosomal protein L23a | P62750 | 0.738 | 0.039 |
| RPL31 | ribosomal protein L31 | P62899 | 0.675 | 0.027 |
| RPL5 | ribosomal protein L5 | P46777 | 1.13 | 0.042 |
| RPS18 | ribosomal protein S18 | P62269 | 0.711 | 0.043 |
| RPS20 | ribosomal protein S20 | P60866 | 0.703 | 0.042 |
| RSU1 | Ras suppressor protein 1 | Q15404 | 2.21 | 0.00082 |
| RTN2 | reticulon 2 | O75298 | 0.35 | 0.016 |
| RTN4 | reticulon 4 | Q9NQC3 | 0.128 | 0.032 |
| SEPTIN2 | septin 2 | Q15019 | 1.22 | 0.042 |
| SH3GLB1 | SH3 domain containing GRB2 like, endophilin B1 | Q9Y371 | 0.707 | 0.049 |
| SNTB1 | syntrophin beta 1 | Q13884 | 0.599 | 0.018 |
| SNX2 | sorting nexin 2 | O60749 | 0.779 | 0.01 |
| SOD1 | superoxide dismutase 1 | P00441 | 1.03 | 0.024 |
| SPTBN1 | spectrin beta, non-erythrocytic 1 | Q01082 | 1.43 | 0.0038 |
| STRAP | serine/threonine kinase receptor associated protein | Q9Y3F4 | 0.614 | 0.009 |
| SYNCRIP | synaptotagmin binding cytoplasmic RNA interacting protein | O60506 | 0.834 | 0.025 |
| TGFBI | transforming growth factor beta induced | Q15582 | 0.976 | 0.039 |
| TGM2 | transglutaminase 2 | P21980 | 0.982 | 0.011 |
| TLN2 | talin 2 | Q9Y4G6 | 0.861 | 0.0053 |
| TPP2 | tripeptidyl peptidase 2 | P29144 | 0.446 | 0.047 |
| TRDN | triadin | Q13061 | 0.6 | 0.0027 |
| TXNDC17 | thioredoxin domain containing 17 | Q9BRA2 | 0.981 | 0.046 |
| TXNL1 | thioredoxin like 1 | O43396 | 0.377 | 0.017 |
| UBE2O | ubiquitin conjugating enzyme E2 O | Q9C0C9 | 1.76 | 0.029 |
| UNC45B | unc-45 myosin chaperone B | Q8IWX7 | 0.933 | 0.033 |
| USP5 | ubiquitin specific peptidase 5 | P45974 | 1.15 | 0.029 |
| VCP | valosin containing protein | P55072 | 0.468 | 0.035 |
| VPS35 | VPS35 retromer complex component | Q96QK1 | 0.544 | 0.032 |

Table S9. Pre-pill and one-hour post-pill plasma sildenafil concentrations (ng/ml) during active treatment with sildenafil (Weeks 0-4 for S-P treatment sequence and Week 8 for P-S treatment sequence). Arithmetic means were used.

| Week | Treatment Sequence | Pre-Pill | | | | One-Hour Post-Pill | | | |
| --- | --- | --- | --- | --- | --- | --- | --- | --- | --- |
|  |  | Mean | SD | Min | Max | Mean | SD | Min | Max |
| Week 0 | S-P | 1.2 | 0.5 | 0.7 | 1.7 | 159.3 | 99.7 | 22.0 | 242.7 |
| Week 4 |  | 9.4 | 7.3 | 3.0 | 18.7 | 324.2 | 167.7 | 159.4 | 506.3 |
| Week 8 | P-S | 14.0 | 12.0 | 2.3 | 29.1 | 177.3 | 62.5 | 91.4 | 225.7 |

Table S10. Pre-pill and one-hour post-pill plasma N-desmethyl sildenafil concentrations (ng/ml) during active treatment with sildenafil (Weeks 0-4 for S-P treatment sequence and Week 8 for P-S treatment sequence). Arithmetic means were used.

| Week | Treatment Sequence | Pre-Pill | | | | One-Hour Post-Pill | | | |
| --- | --- | --- | --- | --- | --- | --- | --- | --- | --- |
|  |  | Mean | SD | Min | Max | Mean | SD | Min | Max |
| Week 0 | S-P | 0.2 | 0.1 | 0.1 | 0.3 | 21.0 | 17.9 | 1.2 | 42.3 |
| Week 4 |  | 1.3 | 0.6 | 0.8 | 2.1 | 33.4 | 10.3 | 18.0 | 38.7 |
| Week 8 | P-S | 1.2 | 0.5 | 0.7 | 1.8 | 14.6 | 9.5 | 2.2 | 25.0 |

Table S11. Pre-pill and one-hour post-pill plasma tadalafil concentrations (ng/ml) during active treatment with tadalafil (Weeks 0-4 for T-P treatment sequence and Week 8 for P-T treatment sequence). Arithmetic means were used.

| Week | Treatment Sequence | Pre-Pill | | | | One-Hour Post-Pill | | | |
| --- | --- | --- | --- | --- | --- | --- | --- | --- | --- |
|  |  | Mean | SD | Min | Max | Mean | SD | Min | Max |
| Week 0 | T-P | 2.2 | 1.8 | 0.5 | 4.6 | 113.9 | 50.0 | 63.3 | 181.6 |
| Week 4 |  | 143.3 | 104.0 | 1.5 | 227.4 | 283.2 | 142.0 | 93.1 | 412.4 |
| Week 8 | P-T | 143.0 | 108.9 | 64.9 | 301.7 | 281.3 | 146.1 | 142.6 | 476.3 |

Table S12. Pre-pill and one-hour post-pill plasma N-desmethyl tadalafil concentrations (ng/ml) during active treatment with tadalafil (Weeks 0-4 for T-P treatment sequence and Week 8 for P-T treatment sequence). Arithmetic means were used.

| Week | Treatment Sequence | Pre-Pill | | | | One-Hour Post-Pill | | | |
| --- | --- | --- | --- | --- | --- | --- | --- | --- | --- |
|  |  | Mean | SD | Min | Max | Mean | SD | Min | Max |
| Week 0 | T-P | 0.0 | 0.0 | 0.0 | 0.1 | 0.0 | 0.0 | 0.0 | 0.1 |
| Week 4 |  | 2.1 | 1.7 | 0.0 | 3.6 | 3.1 | 2.2 | 0.1 | 5.0 |
| Week 8 | P-T | 3.3 | 1.8 | 1.8 | 6.0 | 3.4 | 1.9 | 1.6 | 6.0 |

Table S13. Repeated measures mixed effect model results for daily BFI measure.

|  | **Sildenafil** | | **Tadalafil** | |
| --- | --- | --- | --- | --- |
| **Fixed effects** | p | | p | |
| Time | 0.306 | | 0.071 | |
| **Random effects** | SD | Variance | SD | Variance |
| Subject | 0.953 | 0.909 | 1.346 | 1.811 |
| Residual | 0.926 | 0.858 | 1.067 | 1.139 |
| **Matching** | χ^2^ | p | χ^2^ | p |
|  | 95.36 | <0.0001 | 151.4 | <0.0001 |

Figure S1. Study day timeline for week 0 (top) and weeks 4/8 (bottom). At the week 0 and week 4 study visit, subjects were given the pill matching their treatment for weeks 1-4. During the week 8 study visit, subjects were given the pill matching their treatment for weeks 5-8. Created with BioRender.

Figure S2. Heatmap depicting mapping of upregulated genes to enriched GO Biological Processes term cluster representatives in skeletal muscle following four weeks of sildenafil treatment. Gene names can be found in Table S3. Full heatmap for all terms can be found in Figure S3.

Figure S3. Heatmap depicting mapping of upregulated genes to enriched GO Biological Processes terms in skeletal muscle following four weeks of sildenafil treatment. The gene ratio is the number of upregulated pathway genes in the dataset divided by the total number of genes in the pathway. Gene names can be found in Table S3.

Figure S4. Heatmap depicting mapping of upregulated proteins to enriched GO Biological Processes terms in skeletal muscle following four weeks of sildenafil treatment. Gene symbols are shown. Gene names and UniProt Accession numbers can be found in Table S8.

**Physical performance**

*Handgrip maximum voluntary contraction and endurance*

Long-term effects (pre- to post-treatment differences)

Force and Endurance: Following four weeks of sildenafil treatment, submaximal and maximal voluntary contraction of the dominant arm and submaximal voluntary contraction of the nondominant arm were unchanged (Figure S5A-B). Paired t-tests indicated an increase in overall maximum force of the nondominant arm, which was nonsignificant after correcting for multiple testing (raw p value 0.028, q value 0.736). Following four weeks of tadalafil treatment, submaximal voluntary contraction at 25% effort of the dominant arm decreased, but the effect was nonsignificant following correction for multiple testing (raw p value 0.008, q value 0.874). Submaximal contraction at 50% effort and maximal contraction of the dominant arm, as well as submaximal and maximal voluntary contraction of the nondominant arm at all effort levels were unchanged following treatment. Endurance was unchanged following four weeks of treatment with sildenafil or tadalafil (Figure S5C).

Fatigue: Perceptual fatigue of the dominant and nondominant arms and whole body were unchanged both pre- and post- handgrip testing following four weeks of treatment with sildenafil or tadalafil (Figure S5D-E).

Figure S5. Handgrip force, endurance, and fatigue outcome measures (mean ± SD) prior to and following four weeks of treatment with sildenafil (n=8 for A-C, n=7 for D-E) or tadalafil (n=8).

Short-term effects (pre- to post-pill differences)

Treatment-naïve: Submaximal and maximal voluntary contraction of the dominant and nondominant arm were unchanged one hour post sildenafil dose at treatment-naïve timepoints (Figure S6A-B). Submaximal voluntary contraction of the dominant arm at 25% effort was decreased one hour post tadalafil dose at treatment-naïve timepoints, which was nonsignificant following correction for multiple testing (raw p value 0.041, q value 0.59). Submaximal voluntary contraction at 50% effort and maximal contraction of the dominant arm, as well as submaximal and maximal contraction of the nondominant arm at all effort levels were unchanged one hour post tadalafil dose. Endurance was unchanged one hour post sildenafil or tadalafil dose (Figure S6C).

Treated: Submaximal voluntary contraction of the nondominant arm at 25% effort was decreased one hour post sildenafil dose at treated timepoints, which was nonsignificant following correction for multiple testing (raw p value 0.027, q value 0.590, Figure S6D-E). Submaximal voluntary contraction at 50% effort and maximal contraction of the dominant arm, as well as submaximal and maximal contraction of the nondominant arm at all effort levels were unchanged one hour post sildenafil dose. Submaximal and maximal voluntary contraction of the dominant and nondominant arm were unchanged one hour post tadalafil dose at treated timepoints. Endurance was unchanged one hour post sildenafil or tadalafil dose (Figure S6F).

Figure S6. Handgrip force and endurance outcome measures (mean ± SD) prior to and one-hour post-pill ingestion at A-C) treatment-naïve (n=4 for sildenafil and n=4 for tadalafil) and D-F) treated (n=8 for sildenafil and n=8 for tadalafil) timepoints.

*Leg extension torque, endurance, recovery, and fatigue*

Torque, Endurance, and Recovery: Following four weeks of sildenafil treatment, leg extension isometric and isokinetic peak torque and endurance were unchanged (Figure S7A,D). There was an increase in recovery torque production at 0 minutes post endurance test, but this was nonsignificant after correcting for multiple testing (raw p value 0.038, q value 0.736, Figure S7E). Recovery torque production at 3 and 5 minutes post endurance test were unchanged. Following four weeks of tadalafil treatment, leg extension isometric peak torque was decreased, but this was nonsignificant following correction for multiple testing (Figure S7A). Leg extension peak isokinetic torque, endurance, and recovery were unchanged (Figure S7A,D-E).

Fatigue: Perceived leg and body fatigue assessed pre- and post-Biodex testing were unchanged following four weeks of sildenafil treatment (Figure S7B-C). Following four weeks of tadalafil treatment, there was a decrease in perceived leg fatigue assessed pre-Biodex testing, but this was nonsignificant after correcting for multiple testing (raw p value 0.015, q value 0.736). Perceived leg fatigue assessed post-Biodex testing and perceived body fatigue assessed pre-and post-Biodex testing were unchanged.

*Walking performance*

Average distances walked at 25%, 50%, or 100% perceived effort were unchanged following four weeks of treatment with sildenafil (Figure S7F). Following four weeks of treatment with tadalafil, average distance walked at 25% perceived effort was decreased, although this difference was nonsignificant after correcting for multiple testing (raw p value 0.041, q value 0.736). Average distances walked at 50% or 100% perceived effort were unchanged.

Figure S7. Leg extension torque, endurance, recovery, and fatigue, and walking performance outcome measures (mean ± SD) prior to and following four weeks of treatment with sildenafil (n=4) or tadalafil (n=4).

**Femoral artery blood flow**

Long-term effects (pre- to post-treatment differences)

Common femoral artery blood flow was unchanged following four weeks of treatment with sildenafil or tadalafil (Figure S8).

Figure S8. Common femoral artery blood flow (ml/min, mean ± SD) prior to and following four weeks of treatment with sildenafil (n=7) or tadalafil (n=8).

Short-term effects (pre- to post-pill differences)

Common femoral artery blood flow was unchanged one hour post sildenafil or tadalafil dose at treatment-naïve and treated timepoints (Figure S9A-B).

Figure S9. Common femoral artery blood flow (ml/min, mean ± SD) prior to and one-hour post-pill ingestion at A) treatment-naïve (n=4 for sildenafil and n=4 for tadalafil) and B) treated (n=6 for sildenafil and n=7 for tadalafil) timepoints.

**Body composition**

Lean mass, fat mass, and total mass were unchanged following four weeks of treatment with sildenafil or tadalafil (Figure S10).

Figure S10. Lean mass, fat mass, and total mass (kg, mean ± SD) prior to and following four weeks of treatment with sildenafil (n=8) or tadalafil (n=8).

**Subject-reported outcomes**

POMS and MFSI subscale and total scores were unchanged following four weeks of treatment with sildenafil or tadalafil, with the exception of the POMS Vigor subscale, which was increased following treatment with sildenafil; the effect was nonsignificant after correcting for multiple testing (Figure S11, raw p value 0.025, q value 0.736). Mixed effects analyses indicated no significant change in BFI scores over 4 weeks of treatment with sildenafil or tadalafil (Table S13, Figure S12). Within-subject variability was successfully controlled (p<0.0001) in both models.

Figure S11. A. Profile of Mood States (POMS) subscale and total scores (mean ± SD) and B. Multidimensional Fatigue Inventory – Short Form (MFSI) subscale and total scores (mean ± SD) prior to and following four weeks of treatment with sildenafil (n=8 for POMS and n=6 for MFSI) or tadalafil (n=7). Higher scores are undesirable and indicate greater symptom severity, with the exception of the POMS and MFSI Vigor subscales, for which lower scores indicate greater symptom severity.

Figure S12. Daily Brief Fatigue Inventory (BFI) scores (mean + SD) over four weeks of treatment with sildenafil (n=7) or tadalafil (n=8).

Figure S13. Macrophage counts over four weeks of treatment with placebo for participants assigned to placebo first (weeks 1-4, n=6, paired samples were not available for two subjects due to degraded or insufficient tissue for one or both timepoints).

**Supplementary Discussion**

**Physical performance**

There were no differences in outcomes of muscle strength, fatigue, or endurance in this study. Our previous study found no difference in isokinetic and isometric torque, but endurance was improved following 8 days of treatment with 25 mg sildenafil (Sheffield-Moore et al. 2013). There was also no difference in peak power following an acute dose of 20 mg tadalafil in athletes, although time to peak power was decreased (Guidetti et al. 2008). There was no effect of PDE-5 inhibition on 6-minute walk distances in this study. Previous studies of the effect of PDE-5 inhibitors on the 6-minute walk have had mixed results; improvements were shown in patients with pulmonary hypertension (Falk et al. 2010; Galiè et al. 2009; Lewis et al. 2007) and chronic obstructive pulmonary disease (Rao et al. 2011), but not in patients with idiopathic pulmonary fibrosis (Zisman et al. 2010) or Duchenne’s muscular dystrophy (Witting et al. 2014).

**Blood flow**

In this study, there was no increase in common femoral artery blood flow either following an acute dose or after long-term (4-week) treatment with a PDE-5 inhibitor. One study did observe increased femoral artery blood flow at rest in healthy adults following acute administration of sildenafil, but this was following a higher dose (100 mg) than used in this study (Nyberg et al. 2015). Other studies indicated that forearm blood flow was acutely increased at rest following 20 mg tadalafil administration in Type 2 diabetes patients that were postprandial but not those that were fasted (Sjögren et al. 2016; Jansson et al. 2010), while brachial artery blood flow during exercise was unchanged following long-term treatment with sildenafil (4 weeks, 20 mg 3x per day) in patients with Duchenne’s muscular dystrophy (Witting et al. 2014). Other studies have observed an increase in finger capillary blood flow after long-term low dose (5 mg/day) tadalafil treatment in Type 2 diabetes patients with erectile dysfunction (Lee et al. 2023) and acutely following 50 mg sildenafil in patients with coronary artery disease (Park et al. 2004).

**Patient-reported outcomes**

There were no differences in patient-reported outcomes of fatigue and mood following four weeks of treatment with tadalafil or sildenafil in this study. There is a paucity of studies assessing patient-reported outcomes following PDE-5 inhibitor treatment. Perceptual fatigue and emotional function were unchanged in heart failure patients treated with sildenafil (50 mg 3x per day for 6-12 months) (Zhuang et al. 2014), while depressive symptoms improved in a single-arm pilot study of erectile function patients with depression following long-term low dose treatment with tadalafil (5 mg per day for 8 weeks) (Choi et al. 2019).

**Supplementary References**

Choi, Jin Bong, Kang Jun Cho, Joon Chul Kim, Chi-Un Pae, and Jun Sung Koh. 2019. “An Open-Label, Single-Arm Pilot Study to Evaluate the Efficacy of Daily Low Dose Tadalafil on Depression in Patients with Erectile Dysfunction.” *Translational Andrology and Urology* 8 (5): 501–6.

Falk, Jeremy A., Kiran J. Philip, and Ernst R. Schwarz. 2010. “The Emergence of Oral Tadalafil as a Once-Daily Treatment for Pulmonary Arterial Hypertension.” *Vascular Health and Risk Management* 6 (May): 273–80.

Galiè, Nazzareno, Bruce H. Brundage, Hossein A. Ghofrani, et al. 2009. “Tadalafil Therapy for Pulmonary Arterial Hypertension.” *Circulation* 119 (22): 2894–903.

Guidetti, L., G. P. Emerenziani, M. C. Gallotta, F. Pigozzi, L. Di Luigi, and C. Baldari. 2008. “Effect of Tadalafil on Anaerobic Performance Indices in Healthy Athletes.” *British Journal of Sports Medicine* 42 (2): 130–33.

Jansson, P. A., G. Murdolo, L. Sjögren, et al. 2010. “Tadalafil Increases Muscle Capillary Recruitment and Forearm Glucose Uptake in Women with Type 2 Diabetes.” *Diabetologia* 53 (10): 2205–8.

Lee, Jong Seung, Seung-Ho Hong, Hwa Yeon Sun, et al. 2023. “The Efficacy and Safety of Tadalafil in the Management of Erectile Dysfunction with Diabetes and Blood Circulation Issues.” *The Aging Male: The Official Journal of the International Society for the Study of the Aging Male* 26 (1): 2176484.

Lewis, Gregory D., Ravi Shah, Khurram Shahzad, et al. 2007. “Sildenafil Improves Exercise Capacity and Quality of Life in Patients with Systolic Heart Failure and Secondary Pulmonary Hypertension.” *Circulation* 116 (14): 1555–62.

Nyberg, Michael, Peter Piil, Jon Egelund, Randy S. Sprague, Stefan P. Mortensen, and Ylva Hellsten. 2015. “Potentiation of cGMP Signaling Increases Oxygen Delivery and Oxidative Metabolism in Contracting Skeletal Muscle of Older but Not Young Humans.” *Physiological Reports* 3 (8): e12508.

Park, J. W., C. Mrowietz, N. Chung, and F. Jung. 2004. “Sildenafil Improves Cutaneous Microcirculation in Patients with Coronary Artery Disease: A Monocentric, Prospective, Double-Blind, Placebo-Controlled, Randomized Cross-over Study.” *Clinical Hemorheology and Microcirculation* 31 (3): 173–83.

Rao, Ravinder Singh, Sheetu Singh, Bharat Bhushan Sharma, V. V. Agarwal, and Virendra Singh. 2011. “Sildenafil Improves Six-Minute Walk Distance in Chronic Obstructive Pulmonary Disease: A Randomised, Double-Blind, Placebo-Controlled Trial.” *The Indian Journal of Chest Diseases & Allied Sciences* 53 (2): 81–85.

Sheffield-Moore, Melinda, John E. Wiktorowicz, Kizhake V. Soman, et al. 2013. “Sildenafil Increases Muscle Protein Synthesis and Reduces Muscle Fatigue.” *Clinical and Translational Science* 6 (6): 463–68.

Sjögren, L., J. Olausson, L. Strindberg, et al. 2016. “Postprandial Effects of the Phosphodiesterase-5 Inhibitor Tadalafil in People with Well-Controlled Type 2 Diabetes Mellitus: A Randomized Controlled Trial.” *Diabetic Medicine: A Journal of the British Diabetic Association* 33 (9): 1299–301.

Witting, Nanna, Christina Kruuse, Bo Nyhuus, et al. 2014. “Effect of Sildenafil on Skeletal and Cardiac Muscle in Becker Muscular Dystrophy: Sildenafil in BMD.” *Annals of Neurology* 76 (4): 550–57.

Zhuang, Xiao-Dong, Ming Long, Fei Li, Xun Hu, Xin-Xue Liao, and Zhi-Min Du. 2014. “PDE5 Inhibitor Sildenafil in the Treatment of Heart Failure: A Meta-Analysis of Randomized Controlled Trials.” *International Journal of Cardiology* 172 (3): 581–87.

Zisman, David A., Marvin Schwarz, Kevin J. Anstrom, Harold R. Collard, Kevin R. Flaherty, and Gary W. Hunninghake. 2010. “A Controlled Trial of Sildenafil in Advanced Idiopathic Pulmonary Fibrosis.” *The New England Journal of Medicine* 363 (7): 620–28.
